# Yinchenhao Decoction Ameliorates Alpha-Naphthylisothiocyanate Induced Intrahepatic Cholestasis in Rats by Regulating Phase II Metabolic Enzymes and Transporters

**DOI:** 10.3389/fphar.2018.00510

**Published:** 2018-05-15

**Authors:** Ya-Xiong Yi, Yue Ding, Yong Zhang, Ning-Hui Ma, Feng Shi, Ping Kang, Zhen-Zhen Cai, Tong Zhang

**Affiliations:** ^1^School of Pharmacy, Shanghai University of Traditional Chinese Medicine, Shanghai, China; ^2^Experiment Center for Teaching and Learning, Shanghai University of Traditional Chinese Medicine, Shanghai, China; ^3^Pharmaceutical Preparation Section, Guangming Chinese Medicine Hospital of Pudong New Area, Shanghai, China; ^4^Headmaster’s Office, Shanghai University of Traditional Chinese Medicine, Shanghai, China; ^5^Experiment Center for Science and Technology, Shanghai University of Traditional Chinese Medicine, Shanghai, China

**Keywords:** Yinchenhao Decoction, phase II metabolism, pharmacokinetics, cholestasis, bilirubin metabolism enzyme, metabolic transporter

## Abstract

*Yinchenhao* Decoction (YCHD), a famous traditional Chinese formula, has been used for treating cholestasis for 1000s of years. The cholagogic effect of YCHD has been widely reported, but its pharmacodynamic material and underlying therapeutic mechanism remain unclear. By using ultra-high-performance liquid chromatography (UHPLC)-quadrupole time-of-flight mass spectrometry, 11 original active components and eight phase II metabolites were detected in rats after oral administration of YCHD, including three new phase II metabolites. And it indicated that phase II metabolism was one of the major metabolic pathway for most active components in YCHD, which was similar to the metabolism process of bilirubin. It arouses our curiosity that whether the metabolism process of YCHD has any relationship with its cholagogic effects. So, a new method for simultaneous quantitation of eight active components and four phase II metabolites of rhein, emodin, genipin, and capillarisin has been developed and applied for their pharmacokinetic study in both normal and alpha-naphthylisothiocyanate (ANIT)-induced intrahepatic cholestasis rats. The results indicated the pharmacokinetic behaviors of most components of YCHD were inhibited, which was hypothesized to be related to different levels of metabolic enzymes and transporters in rat liver. So dynamic changes of intrahepatic enzyme expression in cholestasis and YCHD treated rats have been monitored by an UHPLC-tandem mass spectrometry method. The results showed expression levels of UDP-glucuronosyltransferase 1-1 (UGT1A1), organic anion-transporting polypeptide 1A4 (OATP1A4), multidrug resistance-associated protein 2 (MRP2), multidrug resistance protein 1, sodium-dependent taurocholate cotransporter, and organic anion-transporting polypeptide 1A2 were significantly inhibited in cholestasis rats, which would account for reducing the drug absorption and the metabolic process of YCHD in cholestatic rats. A high dose (12 g/kg) of YCHD remarkably increased the expression of UGT1A1, bile salt export pump, MRP2, OATP1A4 in cholestasis rats presented it exhibited the greatest ameliorative effect on cholestasis, also particularly in histopathological examination and reducing levels of alanine transaminase, aspartate transaminase, total bilirubin, direct bilirubin, and total bile acid. Considering the metabolic process of bilirubin *in vivo*, the choleretic effect of YCHD is proven to be related to its regulatory action on expression of metabolic enzymes and transporters in cholestatic liver.

## Introduction

As one of the top 15 causes of death in the United States (Yang et al., 2009), intrahepatic cholestasis is a common clinical pathological process of the liver that can be induced by drugs, hormones, cytokines, stones, and progressive bile duct or liver cell destruction. It increases the risk of liver failure and cirrhosis periductular fibrosis, biliary fibrosis, cirrhosis (Hirschfield et al., 2010), or other hepatic and gall-bladder diseases (Liu et al., 2015). Ursodeoxycholic acid (UDCA) is the only approved drug for treating cholestatic disorders (Paumgartner and Pusl, 2008). However, UDCA overdose can engender side effects such as development of hepatobiliary impairments and cytotoxicity (Chatterjee et al., 2014). Therefore, studying the pathogenesis and treatment of intrahepatic cholestasis is imperative. *Yinchenhao* Decoction (YCHD), a famous traditional Chinese formula, is recorded in the *Shang Han Lun*, a classic traditional Chinese medicine resource compiled by Zhongjing Zhang (150–215 A.D.). YCHD has been used to treat jaundice and liver disorders, including cholestasis, liver fibrosis, hepatitis C, biliary cirrhosis, and cholestatic liver diseases (Zhang et al., 2013). In Japan, it is also known for its ability to inhibit hepatocyte apoptosis as well as promote bile secretion and excretion, known as Inchinkoto or TJ-135. YCHD has been proven to have beneficial effects in promoting hepatic regeneration and preventing post-operative hepatic failure, even for alcoholic liver disease (Wang et al., 2008a). Despite research into its curative effects, the pharmacodynamic material basis and underlying therapeutic mechanism remain unclear.

*Yinchenhao* Decoction is composed of three herbs, *yinchen* (the monarch herb, *Artemisia scoparia* Waldst. et Kitam. or *A. capillaris Thunb*.), *dahuang* (the assistant and servant herb, *Rheum palmatum* L., *R. tanguticum*’ Maxim. ex Balf. or *R. officinale* Baill.), and *zhizi* (the minister herb, *Gardenia jasminoides* Ellis) (Zhang et al., 2013). Constituent chemicals in YCHD such as coumarin, flavone, chromone, anthraquinone, organic acids, triterpene, and steroids have been documented. A previous study in our laboratories developed a method for simultaneously determining the 14 active components of YCHD by using an ultra-high-performance liquid chromatography (UHPLC) system coupled to a diode array detection system, providing researchers insights into the material construction of YCHD. Plasma pharmacochemistry has also been conducted to analyze the potentially effective constituents of YCHD after oral administration, and 21 of the constituents in rat blood were identified through a UHPLC system coupled to a quadrupole time-of-flight tandem mass spectrometry (UHPLC-UV/Q-TOF-MS/MS) system (Wang et al., 2008b). However, previous research mostly focused on the prototype of YCHD’s active constituents, leading to relatively little data on their metabolites Pre-experimental results in our labs showed that major components of YCHD including rhein, genipin, caffeic acid could be bio-transformed to phase II metabolite forms (glucuronidated and sulfated conjugates) immediately, similar to bilirubin metabolism *in vivo*. Therefore, identifying these phase II metabolites and quantifying their level is necessary. However, due to lacking of the corresponding reference substance, the content of phase II metabolites can’t be directly determined. To solve this problem, our previous study (Ding et al., 2013) applied β-glucuronidase and sulfatase to hydrolyze glucuronidated and sulfated conjugates in biological samples into prototypical drugs, thus enabling the determination of phase II metabolite concentrations by measuring the prototype drug indirectly. Thus, pharmacokinetics of phase II metabolites of the active components of YCHD could be conducted using an enzymolysis method in present study, although no standard substance analytes of them existed.

The causes of cholestasis can be extrahepatic, intrahepatic, or both. Intrahepatic cholestasis is caused by inflammatory disorders such as primary biliary cirrhosis, drug toxicity induced by chlorpromazine, anabolic or sex steroids, and others, viral or alcoholic hepatitis. Extrahepatic cholestasis results from obstruction of the bile ducts caused by extraluminal compression, cholelithiasis, or inflammatory processes. And chronic cholestasis can lead to jaundice and hypercholesterolemia, then aggravated outcomes just like clinical signs of liver failure, cirrhosis, and hepatic fibrosis (Boyer, 2007). Furthermore, the hypothesis regarding the mechanisms of drug induced intrahepatic cholestasis have been based on the cell biology of bile production and secretion, including the dysfunction of canalicular motility (Watanabe et al., 1991) and the abnormalities in hepatic membrane transporters (Trauner et al., 1999). Studies have found that the occurrence of cholestasis was accompanied by the modulating of metabolic enzyme and transporters expression like UGT1A1, MRP2, BSEP, OATP1A1, OATP1A4 (Ding et al., 2014; Zhang et al., 2016). As such, research has revealed that protein transporters like MDR1, NTCP, OATP1A2, OCT1, MATE1 play a major role in the metabolism of compounds in the liver (Muller and Fromm, 2011; Nies et al., 2011; Borst and Schinkel, 2013; Koepsell, 2013; Konig et al., 2013; Motohashi and Inui, 2013); thus, the up-regulation and down-regulation of said proteins may show a strong correlation with the metabolism of bilirubin in intrahepatic cholestasis. Most of the constituents of YCHD have the potential to metabolize through glucuronic acid and sulfuric acid conjugation, which was similar to the metabolism process of bilirubin, due to the hydroxyl groups that exist in their structure. Glucuronic acid conjugation is mediated by UGT1A1 and has also been reported to affect the level of UGT1A1 (Wu et al., 2014). On this basis, we suspected that the treatment mechanism of YCHD in cholestatic rats was related to its regulation of bilirubin metabolism through the modulation of drug metabolic enzymes and transporters, which has never been completely investigated in previous studies.

The current study applied an UHPLC-quadrupole time-of-flight mass spectrometry (UHPLC/Q-TOF-MS) method to identify metabolites of YCHD and explore the metabolic profiles of YCHD in rats. Pharmacokinetic differences in active constituents as well as phase II metabolites of YCHD were studied with the aid of UHPLC-MS/MS and enzymatic hydrolysis technology. Furthermore, to explain the pharmacokinetic difference, the expression levels of the metabolic enzyme UGT1A1 and drug transporters MRP2, BSEP, OCT1, NTCP, MATE1, MDR1, OATP1A1, OATP1A2, and OATP1A4 in the liver were evaluated using UHPLC-MS/MS, in control, model, and YCHD-treated groups. Additionally, the ameliorative activity of YCHD at three different doses was studied by investigating the effects on the bile flow rate and levels of serum biochemical indices including alanine transaminase (ALT), aspartate transaminase (AST), total-value bilirubin (TBIL), direct bilirubin (DBIL), and total bile acid (TBA). The aim of this investigation was to reveal whether the choleretic effect of YCHD is related to its regulatory action on bilirubin phase II metabolic enzyme and transporter levels in the liver.

## Materials and Methods

### Chemicals and Reagents

*Yinchen, Dahuang*, and *Zhizi* were supplied by Shanghai Kangqiao TCM, Co., Ltd. (Shanghai, China) and identified by Dr. Y. Ding (Experiment Center for Teaching and Learning, Shanghai University of Traditional Chinese Medicine). The standards geniposidic acid, scandoside methyl ester, chlorogenic acid, genipin-1-β-D-gentiobioside, caffeic acid, geniposide, 4-hydroxyacetophenone, rhein, emodin, and genipin with ≥98% purity were all procured from the National Institute for the Control of Pharmaceutical and Biological Products (Beijing, China). Sulfatase was obtained from Helix pomatia (Type H-1, sulfatase ≥ 10,000 units/g solid, Sigma, United States). The peptides LTIIPQDPILFSGSLR, GVALPETIEEAENLGR, AAATEDATPAALEK, TFQFPGDIESSK, LLLSGFQEELR, STALQLIQR, NTTGALTTR, EENLGITK, SVQPELK, and TYPVPFQR, as well as stable isotope-labeled internal standards (≥98% purity), were synthesized by Bankpeptide Biological Technology, Co., Ltd. (Hefei, China). The ProteoExtract native membrane protein extraction kit was purchased from Calbiochem (Temecula, CA, United States). The BCA protein assay kit and in-solution trypsin digestion kit were obtained from Pierce Biotechnology (Rockford, IL, United States). Formic acid was MS grade; ammonium bicarbonate (98% purity) and sodium deoxycholate (98% purity) were purchased from Sinopharm Chemical Reagent, Co., Ltd. (Shanghai, China). Acetonitrile and methanol, all MS grade, were obtained from Merk (Darmstadt, Germany).

### Preparation and Quality Control of YCHD

*Yinchenhao* Decoction was prepared through the following processes: *yinchen* (18 g), *zhizi* (12 g), and *dahuang* (6 g) were weighed and immersed in 360 mL of distilled water (1:10, w/v) for 12 h. These mixture samples were then boiled twice, each time for 1.5 h. The extraction solutions were mixed and passed through a filter paper. The filtrates were concentrated to 36 mL, and the liquid contained 1 g crude drug/mL. For the quality control (QC) of YCHD and to insure the repeatability of this research, a simultaneous quantification of 14 active components of YCHD was executed using a UHPLC system coupled to a diode array detector (Yi et al., 2016); the 14 components are outlined as follows: geniposidic acid, scandoside methyl ester, chlorogenic acid, genipin-1-β-D-gentiobioside, caffeic acid, geniposide, 4-hydroxyacetophenone, crocin-I, crocin-II, aloe-emodin, rhein, emodin, chrysophanol, and physcion. The quantitation study revealed the major components of YCHD to be geniposide, genipin-1-β-D-gentiobioside, caffeic acid, chlorogenic acid, and rhein, with their content levels being 9274, 2242, 631.7, 280.1, and 260.0 μg/mL, respectively. The dosage of YCHD in this research was calculated as follow formula: Rat Daily Dosage (g/kg) = Adjustment Daily Dosage for Adults (g) × Equivalent Dose Ratio (0. 018 for Human vs. Rat)/Body Weight of Rat (kg) = 33 × 0.018/(200/1000) = 3.2 g/kg. And the adjustment dosages of YCHD (6.0, 9.0, and 12 g/kg) in our research were equal to 2, 3, 4 times of the normal adult dose (Wang et al., 2007) which based on the result of pre-experiment.

### Animals Handing

Male Sprague Dawley rats (200–220 g) were purchased from B&K laboratory Animal, Corp., Ltd. (Shanghai, China), fed in the Laboratory Animal Center of Shanghai University of Traditional Chinese Medicine, and housed in an environmentally controlled animal quarter at a temperature of 22–24°C and a relative humidity of 60–65%. The animals were maintained on a 12:12 h light–dark cycle (lights on at 7:00 am) with regulated temperature and humidity. During the entirety of the experimental process, the rats were fed with certified standard rat chow and tap water *ad libitum*. The rats were divided into three random groups. All efforts were made to reduce animal suffering. Animal experiments strictly complied with the Guide for the Care and Use of Laboratory Animals, and the animal experiment protocols were approved by the Institutional Animal Committee of Shanghai University of Traditional Chinese Medicine (Permit No. SZY201704003).

### Identification of Major Components and Metabolites of YCHD in Rats

The rats in the first group (*n* = 6) were fasted 12 h, and blank blood samples were collected before administration. The rats were orally dosed with YCHD (12 g/kg), and blood samples (200 μL) were collected in heparinized tubes at 5, 15, 30, 45, 60, 120, 240, 360, 480, and 720 min. After centrifuging at 4000 rpm for 10 min, supernatant plasma was harvested into heparinized tubes and then stored at -20°C for preservation. The rats in the second group (*n* = 6) were kept individually in metabolic cages to collect urine and feces. Blank urine and fecal samples were collected at 12 h predosing. Subsequently, the rats were orally administered a single dose of YCHD (12 g/kg of body weight). Urine and fecal samples were collected at 0–24 h after dosing. For the third group of rats (*n* = 6), bile duct intubation was implemented, and blank bile samples were collected before administration. After the administration of YCHD at 12 g/kg of body weight, the drug-containing bile samples were collected.

#### Sample Preparation

Plasma, bile, and urine samples (200 μL each) were separately precipitated with six times of methanol–acetonitrile mixed solution (v/v, 1:1; containing 1% formic acid). After being vortex mixed for 5 min and centrifuged at 15,000 rpm for 5 min at 4°C, the supernatant was separated into another clean tube and dried under a gentle stream of nitrogen at 45°C. Subsequently, the residue was reconstituted in 100 μL of 50% methanol–water (v/v) and then vortex mixed for 10 min. After another round of centrifugation at 15,000 rpm for 5 min at 4°C, 5 μL of the supernatant was analyzed by UHPLC-MS/MS. A 0.5 g sample of naturally dried feces was supplemented with 2 mL of 80% methanol–water (v/v) and then ultrasonically extracted for 30 min at 4°C. After being centrifuged at 15,000 rpm for 10 min, the supernatant was dried under a gentle stream of nitrogen at 45°C. Finally, the residue was reconstituted in 1 mL of 50% methanol–water (v/v) and then vortex mixed for 10 min. After another round of centrifugation at 15,000 rpm for 5 min at 4°C, 5 μL of the supernatant sample was injected into the UHPLC-MS/MS system for analysis.

#### Chromatographic and Mass Spectrometry Conditions

Separations were conducted on an Agilent SB-C18 column (2.1 mm × 50 mm, 1.8 μm) of a UHPLC/Q-TOF-MS system (Waters, Corp., Milford, MA, United States) maintained at 40°C. The column was eluted with 0.4% formic acid in water (A) and acetonitrile (B) as mobile phase under the following gradient conditions: 0–1 min, 5–5% B; 1–4 min, 5–60% B; 4–5 min, 60–70% B; 5–5.1 min, 70–5% B; and re-equilibration for 2 min with 5% B. The flow rate was set at 0.4 mL/min, and the injection volume was 5 μL.

A Synapt G2 quadrupole time-of-flight (Q/TOF) MS/MS system (Waters, Corp., Manchester, United Kingdom) equipped with an electrospray ionization (ESI) interface was operated in negative ionization mode and controlled by MassLynx software (Version 4.1). The optimized conditions for maximum detection of metabolites were as follows: the capillary voltage was set at 3.0 kV, extraction cone voltage was set at 10 and 15 V, source temperature was maintained at 120°C, and desolvation temperature was maintained at 450°C. The cone gas (N_2_) flow rate was set at 50 L/H. Leucine enkephalin was used as the lock mass that generated a reference ion at m/z 554.2651 in positive mode and infused by a lock spray at a flow rate of 5 μL/min for accurate mass acquisition. An MS^E^ approach using a dynamic ramp of collision energy was also applied at two (low and high collision energies) scan functions. The following parameters were set: Function 1 (low energy)—mass scan range, 100–1000; scan time, 0.25 s; interscan delay, 0.05 s; collision energy, 4 V. Function 2 (high energy)—mass scan range, 100–1000; scan time, 0.2 s; interscan delay, 0.05 s; collision energy ramp, 10–30 V. Data were acquired using Masslynx^TM^ (Version 4.1. Waters, Crop., Milford, MA, United States), and Metabolynx^TM^ (Waters, Crop., Milford, MA, United States) was applied for post-acquisition data processing.

### Pharmacokinetic Studies of YCHD in Normal and Cholestatic Rats

#### Instrumentation and Chromatographic Conditions

To explore the pharmacokinetic properties of YCHD following intragastric administration in rats, a rapid and sensitive method involving the use of UHPLC-MS/MS (Agilent 6460 series, Agilent Technologies, Santa Clara, CA, United States) was developed and validated for the simultaneous quantification of 10 active components of rat plasma. The quantification was conducted in ESI negative ionization mode, and the mass spectrometry conditions were as follows: capillary voltage, 3500 V; gas flow, 12 L/min; nebulizer, 40 psi; gas temperature, 350°C; and delta EMV (-), 400. A 5 μL extraction sample was injected into the column (Agilent SB-C18 column, 2.1 mm × 50 mm, 1.8 μm) and eluted at 0.4 mL/min with a gradient elution of water (with 0.4% v/v formic acid) (A) and acetonitrile (B) (0–3 min, 5–60% B; 3–5 min, 60–90% B; 5–5.1 min, 90–5% B; and re-equilibration for 3 min).

#### Sample Preparation

A selective sample preparation method was applied to eliminate the interference of endogenous proteins and to optimize extraction recovery. Among several chemical reagents, methanol, acetonitrile, methanol combined with acetonitrile, and ethyl acetate, ethyl acetate following methanol; moreover, the amount of extraction solvent were all tested in the process. For higher extraction efficiency, 1% formic acid (v/v) was added to the extraction solvent. The application of methanol combined with acetonitrile (v/v, 1:1; containing 1% formic acid) for six times resulted in the highest sensitivity level and convenience, especially in minimizing endogenous interference and enhancing extraction recovery. Therefore, we selected it as the optimal solvent for sample preparation.

To each 50 μL plasma sample, 10 μL of internal standard solution (paeoniflorin, 1000 ng/mL) and 300 μL of a mixed solution of methanol and acetonitrile (v/v, 1:1; containing 1% formic acid) were added. Subsequently, the solution was vortex mixed for 5 min. After centrifugation at 15,000 rpm for 5 min, the supernatant was transferred completely into a tube, before being evaporated under N_2_ at 40°C. The residue was then dissolved in 50 μL of 50% methanol–water (v/v, 1:1) through vortexing for 5 min, after centrifugation at 15,000 rpm for 10 min. Finally, 5 μL of the supernatant sample was injected into the UHPLC-MS/MS system for analysis.

#### Method Validation

**FIGURE 1:**
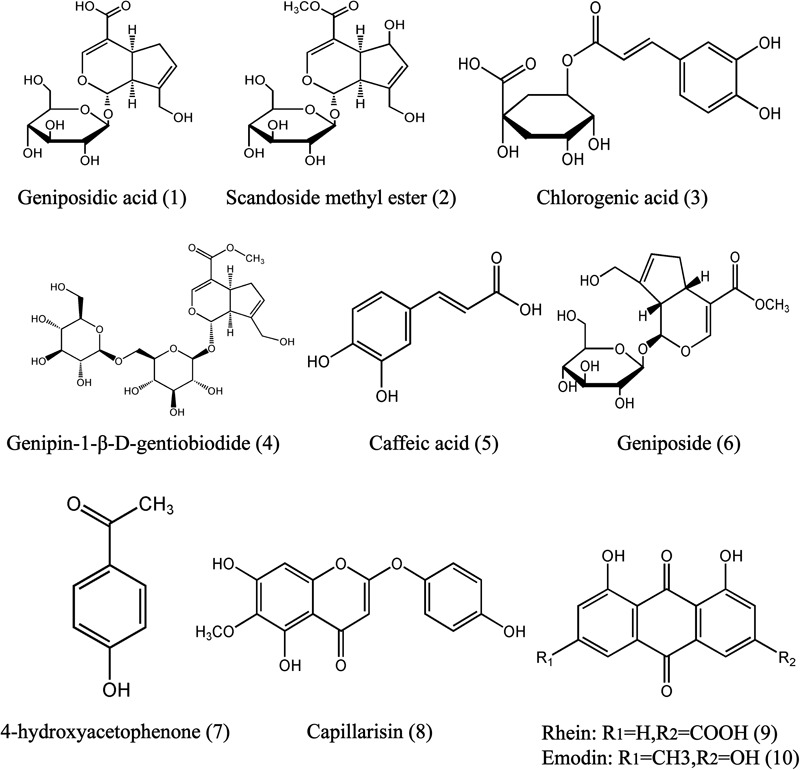
Chemical structures of geniposidic acid (1), scandoside methyl ester (2), chlorogenic acid (3), genipin-l-β-gentiobiodide (4), caffeic acid (5), geniposide (6), 4-hydroxyacetophenine (7), capillarisin (8), and rhein (9), emodin (10) in YCHD.

The UHPLC-MS/MS method developed for determining the 10 components in rat plasma (**Figure 1**)—namely genipin-1-β-D-gentiobiodide, scandoside methyl ester, geniposide, geniposidic acid, chlorogenic acid, capillarisin, rhein, emodin, caffeic acid, and 4-hydroxyacetophenone—was validated in terms of specificity, accuracy, precision, linearity, lower limit of quantitation (LLOQ), stability, recovery, and matrix effect, in accordance with United States Food and Drug Administration guidelines. Calibration curves were constructed using the peak area ratios of the analytes to peoniflorin and by applying a weighted (1/x^2^) least squares linear regression analysis. The LLOQ was determined at the lowest concentrations at which the signal-to-noise (S/N) ratio was 10. Specificity was determined by comparing chromatograms of blank rat plasma obtained from five individual subjects with chromatograms of plasma samples obtained after YCHD administration at a dose of 12 g/kg. Precision [expressed as the relative standard deviation (RSD)] and accuracy [expressed as the relative error (RE)] were calculated for three QC points (low, medium, and high). Five replicates of each QC point were analyzed to determine the interday accuracy and precision. This process was repeated three times over three consecutive days to determine the intraday accuracy and precision. Recovery was evaluated in five replicates at three different QC concentrations (low, medium, and high). The percentage recovery was determined by comparing the concentrations of the pre-extraction spiked QC samples prepared in blank matrix (by adding analytes and peoniflorin to blank matrix prior to extraction) with the peak area of the post-extraction spiked QC samples prepared in an extracted blank matrix (prepared by adding analytes and peoniflorin to blank matrix extract). Matrix effects were investigated on five independent sources of blank rat plasma by calculating the ratio of the peak area in the presence of matrix to the peak area in absence of matrix at three different QC concentrations (low, medium, and high).

The stability of standard analytes in rat plasma was evaluated under various conditions (time and temperature) by analyzing five replicates of the QC samples at three concentrations (low, medium, and high). Stability was investigated in terms of short-term and long-term stability, freeze and thaw stability, and post-preparative stability by using the developed method. Short-term stability was evaluated by storing QC samples at room temperature (25°C) for 24 h. Long-term stability was assessed after 60 days of storage at -20°C. Freeze and thaw stability was determined after three freeze–thaw cycles at -20°C. In addition, post-preparative stability during storage in an auto sampler at 4°C for 24 h was investigated.

#### Application to Pharmacokinetic Analysis

The rats were randomly divided into two groups (*n* = 5 for each group). Group 1 was treated with physiological saline (0.9%) at a dose of 1 mL/100 g, whereas group 2 was treated with ANIT at a dose of 50 mg/kg (diluted in olive oil) to induce cholestatic liver injury. After 24 h, the two groups of rats received a single oral dose of YCHD (12 g/kg). Blood was collected in heparinized tubes at 5, 15, 30, 45, 60, 120, 240, 360, 480, 720, and 1440 min after administration. The blood samples were centrifuged at 4000 rpm for 10 min, and the supernatant plasma was harvested. Plasma samples (50 μL) were immediately processed as outlined in Section “Chromatographic and Mass Spectrometry Conditions.”

Additional plasma samples (50 μL) were enzymatically hydrolyzed with 50 μL of enzyme solution (65.86 mg of sulfatase, dissolved in 2 mL of sodium acetate buffer, pH 5.0) for phase II metabolite quantification, in accordance with our previous study (Ding et al., 2012). After being vortex mixed for 5 min, the mixture was incubated at 37°C for 30 min and then returned to room temperature. Subsequently, the samples were processed as outlined in Section “Chromatographic and Mass Spectrometry Conditions” for UHPLC-MS/MS analysis. During routine analysis, each analytical run included six blank plasma samples, a set of calibration samples, a set of QC samples, and unknowns.

### Protective Effect of YCHD on ANIT-Induced Cholestatic Liver Injury

**FIGURE 2:**
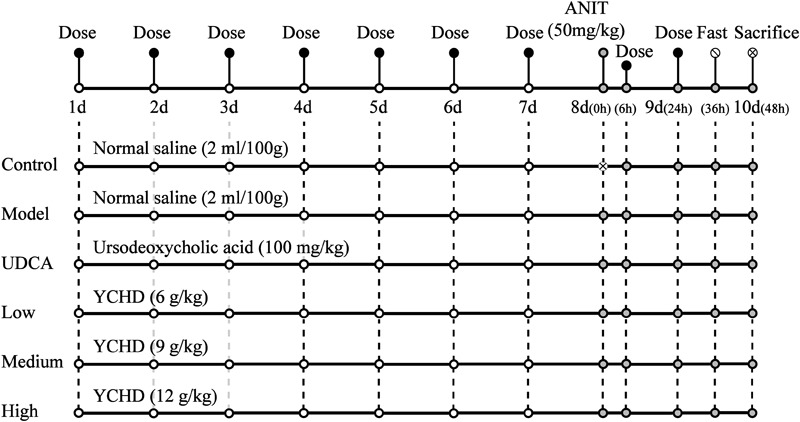
The scheme of the animal experimental design. Control, control group treated with normal saline and then without ANIT; Model, model group treated with normal saline and then with ANIT (50 mg/kg, i.g.); UDCA, positive control group treated with ursodeoxycholic acid (UDCA) (100 mg/kg, i.g.) and ANIT (50 mg/kg, i.g.); Low, Medium, High group rats treated with YCHD at doses of 6.0, 9.0, 12 g/kg, and ANIT (50 mg/kg, i.g.) respectively, “x” in the figure represents rats which were not be treated with ANIT.

**Figure 2** shows the scheme of animal experimental. Male Sprague Dawley rats (230 ± 20 g, 6–8 weeks of age) were purchased from B&K Laboratory Animal, Corp. Ltd. (Shanghai, China) and fed in the Laboratory Animal Center of Shanghai University of Traditional Chinese Medicine. All rats were allowed to acclimatize for 3 days before experimentation. The rats were randomly divided into six groups (*n* = 5 for each group). The rats in group 1 served as non-treated controls, group 2 served as an ANIT-induced cholestasis model, group 3 served as a UDCA-treated (concentration, 10 mg/mL) model, group 4 served as a low-dose YCHD-treated (6 g crude drug/mL) model, group 5 served as a medium-dose YCHD-treated (9 g crude drug/mL) model, and group 6 served as a high-dose YCHD-treated (12 g crude drug/mL) model. The rats in groups 4, 5, and 6 were intragastrically administered YCHD every 24 h for 10 consecutive days. The rats in group 3 were orally administered UDCA at a dose of 100 mg/kg every 24 h for 10 consecutive days, which is well-documented to treat induced liver injury and cholestasis (Chen et al., 2016; Cao et al., 2017). Moreover, groups 1 and 2 were intragastrically treated with physiological saline (0.9%) at a dose of 1 mL/100 g. On the eighth day, all the groups, except for group 1, were treated with ANIT at a dose of 50 mg/kg to induce cholestatic liver injury. Before and 24 h after modeling, retro-orbital blood samples were collected in a coagulation tube, and they were then centrifuged at 4°C for 10 min (4000 rpm) to obtain the serum for the assay of ALT, AST, TBIL, DBIL, and TBA. At the end of the experiment, all rats were euthanized by CO_2_ inhalation. The serum samples were stored at -70°C until analysis, and liver samples were isolated and stored at -70°C for further analysis, whereas the central part of the right large lobe of the liver was used for histological examination.

#### Bile Flow Rate and Serum Biochemistry Analysis

A closed IV catheter system (Becton Dickinson, United States) was applied to cannulate the bile duct, and the bile specimens were collected into tubes at 2 h intervals for 6 h. The collected serum samples were maintained at room temperature to dissolve. The serum levels of ALT, AST, TBIL, DBIL, and TBA were then determined using a chemistry analyzer system (HITACHI 7080, Japan).

#### Determination of UGT1A1 and Nine Other Transporter Proteins in the Liver Using UHPLC-MS/MS

Biotransformation of xenobiotics and endogenous compounds is a crucial step in the absorption and elimination of potentially harmful substances from the human body, a process that could be affected by the levels of pharmacokinetic enzymes and transporter proteins. On the basis of the metabolic process of bilirubin, UGT1A1 and nine other transporter proteins (MRP2, BSEP, OCT1, NTCP, MATE1, MDR1, OATP1A1, OATP1A2, and OATP1A4) were used to evaluate the effect of YCHD on cholestatic rats. Previous methods of protein quantification are immunological, such as immunoblotting (Zhang et al., 2016), reverse transcription polymerase chain reaction (Chen et al., 2016; Lin et al., 2017), and two-dimensional electrophoresis (Bruschi et al., 2015). However, these methods require antibodies that are frequently unavailable; even if available, they are often of uncertain specificity. Furthermore, these assays are characterized by poor reproducibility and accuracy, a narrow analytical range, and low sample throughput. A milestone in quantitative protein analysis was the introduction of mass spectrometry-based analysis using shotgun proteomic assays with relative protein quantification, which has advantages in terms of absolute accuracy, stability, operability, and efficiency over traditional quantitative methods (Wang et al., 2015).

##### Peptide selection

Peptide sequences of the UGT1A1 and transporter proteins were obtained from the UniProt database. An outline of the selection process is as follows:

(1) Peptides of choice were 9 to 16 amino acid residues that were not embedded in the membrane and did not contain any known polymorphic variants or post-translational modifications.
(2) No degradable peptides were selected (e.g., containing cysteine residue or methionine).
(3) Successive R and K sequences (RR, RK, KR, and KK) were excluded to avoid miscleavage of trypsin.
(4) Peptides must have been tryptic with no leaky linkage.
(5) Selected peptides were chemically stable and had ionization efficiency.

The sequence of characteristic peptides that corresponded to UGT1A1 and the other nine transporter proteins are listed in **Table 3**.

##### Sample preparation

Total membrane protein was isolated (in triplicate) from liver tissue according to the Native Membrane Protein Extraction Kit protocol. Subsequently, protein concentrations were determined by applying the BCA Protein Assay Kit. The preparation process was undertaken as outlined in Wang’s research (Wang et al., 2015), with minor adjustments. Specifically, 10 μL of 5.0 mg/mL (or lower concentration) of a hepatocyte membrane protein was incubated with 20 μL of dithiothreitol (100 mM) and 50 μL of ammonium bicarbonate buffer (50 mM, pH 7.8). After incubation at 95°C for 5 min, 20 μL of iodoacetamide (200 mM) was added to the mixture, followed by incubation at room temperature for 20 min in the dark. To concentrate the samples, ice-cold methanol (0.5 mL), chloroform (0.2 mL), and water (0.2 mL) were added to each sample. After centrifugation at 4°C for 5 min at 16,000 *g*, the pellet was washed once with ice-cold methanol (0.5 mL) and resuspended with 40 μL of reconstitution solution [equal volume of 3% sodium deoxycholate (w/v) and 50 mM ammonium bicarbonate buffer]. Finally, the protein sample was digested with 20 μL of trypsin. The protein-to-trypsin ratio was 25:1 (w/w). After incubation at 37°C for 24 h, the digestion reaction was quenched by 30 μL of labeled peptide internal standard (SIL) cocktail (prepared in 15% acetonitrile in water). The samples were centrifuged at 5000 *g* for 5 min at 4°C, and 5 μL of the supernatant was introduced to the UHPLC-MS/MS system.

##### Instrumentation and conditions

An Agilent 1290 Infinity series UHPLC system coupled to an Agilent 6460 series MS/MS system (Agilent Technologies, Santa Clara, CA, United States) was applied for quantitation of the signature peptides in ESI positive ionization mode. The mass spectrometry conditions were as follows: capillary voltage, 2000 V; gas flow, 8 L/min; nebulizer, 30 psi; gas temperature, 300°C; delta EMV (+), 400. A 5 μL digest sample was injected into the column (Agilent SB-C18 column, 2.1 mm × 50 mm, 1.8 μm) and eluted at 0.4 mL/min with a gradient elution of water (with 0.5% v/v formic acid) (A) and acetonitrile (B) (0–1 min, 5–5% B; 1–4 min, 5–60% B; 4–5 min, 60–5% B; and re-equilibration for 3 min).

##### Method validation

The internal standard method was validated in terms of LLOQ, linearity, accuracy, precision, and stability. Calibration curves and QC samples were generated using extraction buffer II (EB II) as blank matrix. Eight calibrators were used to generate 10 calibration curves, which ranged from 0.221 to 70.72 nM. Three QC levels (low, medium, and high concentrations) were applied to validate the UHPLC-MS/MS method. For LLOQ determination, the solutions of the signature peptides were further diluted with EB II to produce a series of concentrations. LLOQ values under optimal chromatographic and MS conditions were determined at an S/N of 10. To evaluate the intraday and interday precision, the QC samples were analyzed in five replicates once a day for three consecutive days. In addition, accuracy and stability (room temperature, freeze and thaw, and autosampler conditions) tests were conducted.

For metabolic enzymes and transport proteins that are endogenous in the rat liver, a method of standard addition was employed to perform recovery. Three concentrations of the 10 standard peptide analytes were prepared (approximately equal to 120, 100, and 80% of the typical concentration of peptides in a normal rat liver digestive sample); the three concentrations were then added to the known samples. Spiked samples were analyzed in three replicates for each concentration. The mean recovery was calculated using the following formula: recovery (%) = 100% × (amount found – original amount)/amount spiked.

#### Metabolic Enzyme and Transporter Level Analysis

The UHPLC–MS/MS absolute quantification method was further applied to determine the expression levels of the metabolic enzymes and transporters (fmol/μg protein) of the 10 proteins in livers among the six groups of rats. The concentration was calculated using the following formula:

$$\text{C}\;=\;\frac{{\text{C}}_{\text{m}}\times \text{V}}{{\text{M}}_{\text{sp}}\times {\text{m}}_{\text{p}}}\;\;{\text{m}}_{\text{p}}\;=\;{\text{C}}_{\text{p}}\;\times \;{\text{V}}_{\text{p}}$$

where C_m_ denotes the measured concentration of metabolic enzymes and transporters, as determined by UHPLC-MS/MS, and V denotes the volume of the final digestive system. Moreover, M_sp_ represents the relative molecular mass of signature peptides of the corresponding metabolic enzymes and transporters; m_p_ and C_p_ represent the mass and concentration of total proteins in the membrane protein, respectively; and V_p_ represents the volume of the membrane protein used for digestive reaction.

### Data Processing and Statistical Analysis

All data are presented as mean ± SD. A one-way analysis of variance was used to compare data between two groups using SPSS (Version 21.0). The differences were considered to be statistically significant when *p* < 0.05 and highly significant when *p* < 0.01 or *p* < 0.001. Pharmacokinetic data analysis was performed using DAS 2.1.1 software (Mathematical Pharmacology Professional Committee of China, Shanghai, China).

## Results

### Phase II Metabolites Identification of YCHD in Rats

**FIGURE 3:**
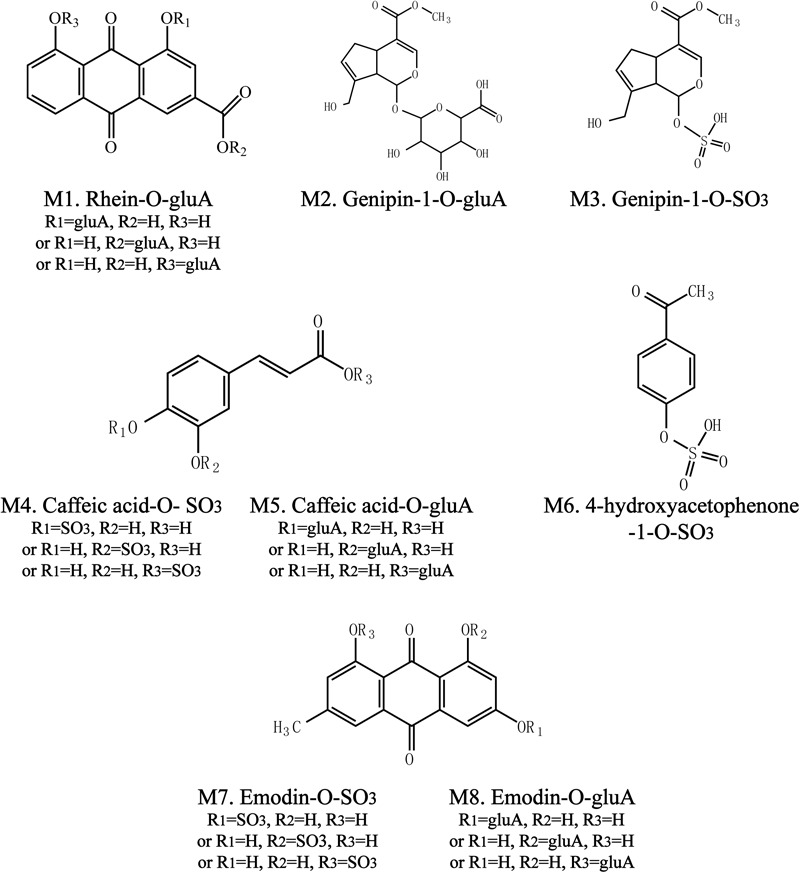
Chemical structure of phase II metabolites of rhein (Ml), genipin (M2, M3), caffeic acid (M4, M5), 4-hydroxyacetophenone (M6), and emodin (M7, M8) after oral adminitration of YCHD (12 g/kg B.W.).

To identify the potential bioactive constituents in rats after oral administration of YCHD, the metabolic profile of YCHD was systematically analyzed using the UHPLC-Q/TOF-MS method. The UHPLC and MS conditions were optimized to obtain a full overview of metabolites by comparing drug-containing biological samples with blank biological samples. First, the chromatographic and MS fragmentation behaviors of the parent compound in YCHD were investigated. Subsequently, metabolite structures were identified by comparing the fragment ion spectra of the parent compound with those of its metabolites. In total, 11 original forms of bioactive components and 19 metabolites including 8 phase II metabolites were detected and structurally identified. The retention times and fragment ions, chemical structure of these metabolites are summarized in **Figure 3** and **Table 1**. The maximum mass errors between the measured and calculated values were determined to be <8 ppm, indicating a high level of confidence. The possible metabolic pathways of the bioactive components of YCHD include demethylated, ring-opened, hydroformylated, glucuronidated, and sulfated transformations. Furthermore, sulfated and glucuronidated conjugates were identified as the major metabolites of rhein, genipin, caffeic acid, 4-hydroxyacetophenone, and emodin. Genipin and emodin were only presented in their phase II forms, and this is because the hydroxyl and phenolic hydroxyl groups in their structure can easily be conjugated by glucuronic acid and sulfuric acid. The Supplementary Figure S1 shows identification chromatogram of eight phase II metabolites of YCHD in rat.

**Table 1:** Identified metabolites of rhein, emodin, caffeic acid, genipin, and 4-hydroxyacetophenone of YCHD in rat.

No.	t_R_ (min)	Identified compound	Mass	Error	Formulate	MS/MS fragment ions (m/z)	Source
			(neutral)	(ppm)			
Data from the ESI-mode		
O1	3.86	Rhein	284.0321	-0.7	C_15_H_8_O_6_	283.0243 [M-H]^-^, 239.0344 [M-H-CO_2_]^-^, 211.0396 [M-H-CO_2-_-CO]^-^, 193.0290 [M-H-CO_2_-CO-H_2_O]^-^, 183.0446[M-H-CO_2_-C_2_O_2_]^-^, 139.0548 [M-H-CO_2_-CO-CO-CO2]^-^	Plasma, bile, urine, feces
O2	2.13	Genipin	226.0841	2.7	C_11_H_14_O_5_	225.0763 [M-H]^-^, 207.0657 [M-H-H_2_O]^-^, 175.0395 [M-H-H_2_O-CH_4_O]^-^, 147.0446 [M-H-H_2_O-CH_4_O-CO]^-^, 123.0446 [M-H-CH_4_O-C_4_H_6_O_3_]^-^, 101.0239 [M-H-CH_4_O-C_7_H_8_O_2_]^-^	Bile, urine, feces
O3	2.10	Caffeic acid	180.0423	0.6	C_9_H_8_O_4_	135.0446 [M-H]^-^, 121.0290 [M-H-CO_2_]^-^	Plasma, bile, urine, feces
O4	2.37	4-Hydroxyacetophenone	136.0524	-1.5	C_8_H_8_O_2_	135.0446 [M-H]^-^, 121.0290 [M-H-CH_2_]^-^	Plasma, bile, urine, feces
O5	4.31	Emodin	270.0528	0.4	C_15_H_10_O_5_	269.0450 [M-H]^-^, 241.0501 [M-H-CO]^-^, 225.0552 [M-H-CO_2_]^-^, 197.0603 [M-H-CO-CO_2_]^-^	Plasma, bile, urine, feces
O6	1.89	Scandoside methyl ester	404.1319	0.5	C_17_H_24_O_11_	403.1240 [M-H]^-^, 371.0978 [M-H-CH_4_O]^-^, 241.0712 [M-H-C_6_H_10_O_5_]^-^, 223.0606 [M-H-C_6_H_10_O_5_-H_2_O]^-^, 213.0763 [M-H-C_6_H_10_O_5_-CO]^-^, 205.0501 [M-H-C_6_H_10_O_5_-H_2_O-H_2_O]^-^, 139.0395 [M-H-C_6_H_10_O_5_-C_4_H_6_O_3_]^-^, 101.0239 [M-H-C_6_H_10_O_5_-C_7_H_8_O_3_]^-^	Plasma, bile, urine, feces
O7	2.14	Geniposide	388.1369	-1.0	C_17_H_24_O_10_	387.1291 [M-H]^-^, 225.0763 [M-H-C_6_H_10_O_5_]^-^, 207.0657 [M-H-C_6_H_10_O_5_-H_2_O]^-^, 175.0395 [M-H-C_6_H_10_O_5_-H_2_O-CH_4_O]^-^, 147.0446 [M-H-C_6_H_10_O_5_-H_2_O-CH_4_O-CO]^-^, 123.0446 [M-H-C_6_H_10_O_5_-C_4_H_6_O_3_]^-^, 101.0239 [M-H-C_6_H_10_O_5_-C_7_H_8_O_2_]^-^	Plasma, bile, urine, feces
O8	1.02/1.06	Geniposidic acid	374.1213	-1.9	C_16_H_22_O_10_	373.1128 [M-H]^-^, 211.0558 [M-H-C_6_H_10_O_5_]^-^, 167.0704 [M-H-C_6_H_10_O_5_-C0_2_]^-^, 193.0464 [M-H-C_6_H_10_O_5_-H_2_O]^-^, 123.0445 [M-H-C_6_H_10_O_5_-C_3_H_4_O_3_]^-^	Plasma, urine, feces
O9	2.1	Genipin-1-β-D-gentiobiodide	549.1819	-1.3	C_23_H_34_O_15_	549.1812 [M-H]^-^, 225.0772 [M-H-2C_6_H_10_O_5_]^-^, 207.0673 [M-H-C_6_H_10_O_5_-H_2_O]^-^, 147.0469 [M-H-C_6_H_10_O_5_-H_2_O-CH_4_O-CO]^-^, 123.0437 [M-H-C_6_H_10_O_5_-C_4_H_6_O_3_]^-^, 101.0251 [M-H-C_6_H_10_O_5_-C_7_H_8_O_2_]^-^	Bile, urine
O10	2.44	Capillarisin	316.0583	7.6	C_16_H_12_O_7_	315.0529 [M-H]^-^, 301.0380 [M-H-CH_2_]^-^, 283.0242 [M-H-CH_2_-H_2_0]^-^	Urine, feces
O11	1.98	Chlorogenic acid	354.0951	-1.4	C_16_H_18_O_9_	353.0868 [M-H]^-^, 191.0557 [M-H-C_11_H_6_O_3_]^-^, 173.0456 [M-H-C_11_H_6_O_3_-H_2_O]^-^, 179.0368 [M-H-C_7_H_10_O_5_]^-^, 135.0454 [M-H-C_7_H_10_O_5_-CO_2_]^-^	Urine
M1	2.61	Rhein-*O*-gluA	460.0642	3.9	C_21_H_16_O_12_	459.0564 [M-H]^-^, 239.0344 [M-H-C_6_H_8_O_6_]^-^, 283.0243 [M-H-C_6_H_8_O_6_-CO_2_]^-^	Urine
M2	2.08	Genipin-1-*O*-gluA	402.1162	-6.2	C_17_H_22_O_11_	401.1084 [M-H]^-^, 225.0763 [M-H-C_6_H_8_O_6_]^-^, 207.0657 [M-H-C_6_H_8_O_6_-H_2_O]^-^, 147.0446 [M-H-C_6_H_8_O_6_-C_2_H_4_O_2_]^-^, 123.0446 [M-H-C_6_H_8_O_6_-C_4_H_6_O_3_]^-^, 101.0239 [M-H-C_6_H_8_O_6_-C_7_H_8_O_2_]^-^	Plasma, bile, urine, feces
M3	1.98	Genipin-1-*O*-SO_3_	305.0335	1.3	C_11_H_14_SO_8_	305.0331 [M-H]^-^, 225.0763 [M-H-SO_3_]^-^, 207.0657 [M-H-SO_3_-H_2_O]^-^, 123.0446 [M-H-SO_3_+H_2_O-C_4_H_6_O_3_]^-^	Bile, urine, feces
M4	1.04/1.80	Caffeic acid-*O*-SO_3_	258.9922	3.9	C_9_H_8_SO_7_	258.9912 [M-H]^-^, 179.0344 [M-H-SO_3_]^-^, 135.0446 [M-H-SO_3_-CO_2_]^-^	Urine, feces
M5	1.85	Caffeic acid-*O*-gluA	355.0674	2.5	C_15_H_16_O_10_	355.0665 [M-H]^-^, 179.0344 [M-H-C_6_H_8_O_6_]^-^, 135.0446 [M-H-C_6_H_8_O_6_-CO_2_]^-^	Plasma, bile, urine
M6	1.78	4-Hydroxyacetophenone-1-*O*-SO_3_	215.0017	1.4	C_8_H_8_SO_5_	215.0014 [M-H]^-^, 135.0446 [M-H-SO_3_]^-^, 121.0290 [M-H-SO_3_-CH_2_]^-^	Plasma, bile, urine, feces
M7	2.84	Emodin-*O*-SO_3_	349.0011	-2.0	C_15_H_9_SO_8_	349.0018 [M-H]^-^, 269.0450 [M-H-SO_3_]^-^, 225.0552 [M-H-SO_3_-CO_2_]^-^	Plasma, urine, feces
M8	2.34	Emodin-*O*-gluA	445.0779	1.8	C_21_H_17_O_11_	445.0771 [M-H]^-^, 269.0450 [M-H-C_6_H_8_O_6_]^-^	Plasma, bile, urine, feces

### Pharmacokinetics Study on the Major Components and Phase II Metabolites

Based on the identified metabolites, a simultaneous determination method for 11 bioactive compounds and four phase II metabolites was developed using UHPLC-MS/MS in multiple-reaction monitoring mode (**Table 2**), along with enzymatic hydrolysis technology. The method was evaluated by comparing chromatograms of six extracted blank plasma samples from different rats with those of spiked plasma samples containing 11 compounds and peoniflorin. The QC samples spiked with 11 compounds and peoniflorin and the real subject’s plasma sample chromatograms collected at 15 min after the administration of YCHD by oral gavage are presented in Supplementary Figures S2–S5. Under optimal chromatographic and mass spectrometer conditions, excellent linearity was achieved for all investigated components, with the square of correlation coefficients (R^2^) being greater than 0.9812 and LLOQ ranging from 0.21 to 7.20 ng/mL (Supplementary Tables S1, S6). The interday precision and intraday precision were in the ranges of 0.6–16.7 and 2.6–16.8% at all QC levels, respectively. Accuracy was calculated as RE (relative error percentage) % = (measured samples/spiked plasma – 1) × 100%l; the derived relative errors ranged from -10.64 to 16.25% (Supplementary Tables S2, S7). The results of matrix effect and extraction recovery of all components at three concentrations are shown in Supplementary Tables S3, S4, S8, and S9. The recovery range was from 47.4 to 101.9% at low, medium, and high concentrations for total constituents (RSD < 17.0%), and the absolute matrix effect values ranged from 44.8 to 116.2%, with the RSD value being lower than 15.6%. The results indicated no coeluting peaks, which may have influenced the ionization of all components and peoniflorin. Finally, stability was assessed using all bias values of the calculated concentrations between post-storage and initial QC samples, and it ranged from 71.57 to 129.2% (Supplementary Tables S5, S10). The results indicate that the 11 compounds were relatively stable under routine laboratory conditions and that no specific procedure was required to stabilize the compounds for pharmacokinetic study.

**Table 2:** Multiple reaction monitoring parameters of ten constituents, genipin, and internal standard.

Component	Parent ion	Product ion	Fragmentor	Collision energy (V)
Genipin-1-β-D-gentiobiodide	595.0	548.9	130	7
Scandoside methyl ester	449.2	241.0	110	10
Geniposide	433.0	225.0	110	10
Geniposidic acid	373.0	122.8	150	19
Chlorogenic acid	353.0	191.0	100	14
Capillarisin	315.1	300.1	140	20
Rhein	283.0	238.9	90	8
Emodin	269.0	225.0	180	24
Caffeic acid	179.1	135.0	90	16
4-Hydroxyacetophenone	134.9	91.9	110	25
Genipin	225.1	123.1	50	12
Paeoniflorin (IS)	479.3	449.2	160	6

**FIGURE 4:**
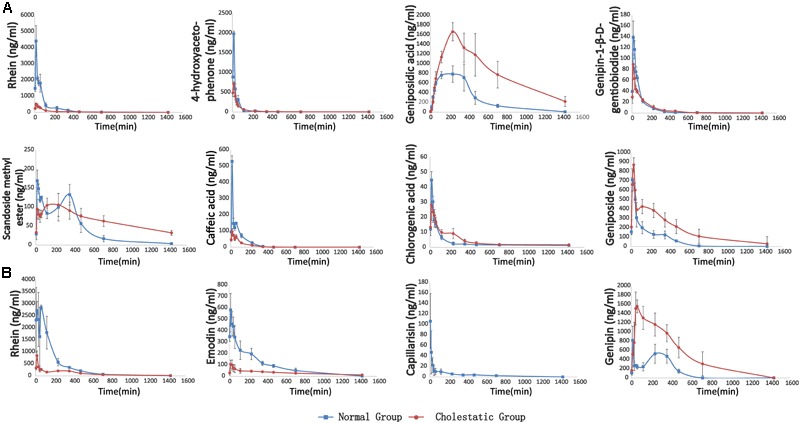
**(A)** The pharmacokinetic curves of each components in normal and ANIT-induced (50 mg/kg) cholestatic rats, which were treated with YCHD at dose of 12 g/kg i.g., and the serums were not treated with sulfatase. **(B)** The pharmacokinetic curves of phase II metabolites of rhein, emodin, capillarisin and genipin, respectively. The data was the difference between the content of post-enzymolysis and pre-enzymolysis.

Following the administration of YHCD to the rats, the UHPLC-MS/MS method was successfully used in the pharmacokinetic study of eight components, because emodin, genipin, and capillarisin had been transformed into phase II metabolites completely and could not be detected directly. The mean plasma concentration-time profiles of genipin-1-β-D-gentiobiodide, scandoside methyl ester, geniposide, geniposidic acid, chlorogenic, rhein, caffeic acid, and 4-hydroxyacetophenone in normal and cholestatic rats are shown in **Figure 4A**, indicating that the pharmacokinetic behaviors of the majority of YCHD components are significantly different in normal rats when compared with cholestatic model rats. The C_max_ values of geniposid, geniposidic acid, and caffeic acid in cholestatic model rats increased by 10.2, 153.7, and 138.8%, respectively, and the AUC_(0-∞)_ values increased by 148.9, 85.5, and 191.3%, respectively, compared with those observed in normal rats. By contrast, the C_max_ values of rhein, 4-hydroxyacetophenone, genipin-1-β-D-gentiobiodide, scandoside methyl ester, and caffeic acid were reduced by 86.1, 57.2, 39.5, 26.3, and 72.4%, respectively, and the AUC (0-∞) values were reduced by 80.9, 41.2, 17.7, -28.8, and 48.9%, respectively. A comparison of Ka and Ke indicated that the *in vivo* absorption and excretion processes of rhein, 4-hydroxyacetophenone, scandoside methyl ester, and caffeic acid were inhibited in the cholestatic rats, whereas the absorption and excretion of geniposide and geniposidic acid were significantly enhanced.

The metabolism study indicated that rhein, emodin, 4-hydroxyacetophenone, caffeic acid, and aglycone of genipin-1-β-D-gentiobiodide and geniposide transformed into their phase II metabolites in rats. Moreover, the aglycone of genipin-1-β-D-gentiobiodide and geniposide, genipin, and emodin could not be directly detected because they were immediately and completely transformed into phase II metabolites. Although these phase II metabolites can be excreted through bile and urine, they can still play a critical role as a major substance influencing efficacy. For example, phase II metabolites of emodin have been reported to have anti-inflammatory and antioxidant effects (Shia et al., 2010, 2011). Baicalin, as a natural baicalein glucuronide, can be metabolized into a variety of baicalein glucuronide conjugates and can protect against fatty liver and liver fibrosis. The metabolic pathways of these compounds are associated with bilirubin metabolic pathways and might affect the key enzymes and transporter proteins of bilirubin metabolism (Guo et al., 2009; Qiao et al., 2011). Therefore, studying the metabolic process of these metabolites *in vivo* is crucial. Consequently, we used biological enzymolysis combined with UHPLC-MS/MS technology to detect these compounds, the glucuronidated and sulfated conjugates of rhein, emodin, genipin, and capillarisin.

After sulfatase-aided enzymolysis, all the phase II metabolites could transform into their prototypes, including rhein, emodin, genipin, and capillarisin. The specific contents of the phase II metabolites of these components were detected indirectly by evaluating the content difference of their prototypes in samples after enzymolysis and before enzymolysis

$${\text{C}}_{\text{G}}\;=\;{\text{C}}_{\text{a}}\;-[{\text{M}}_{\text{G}}\;\times \;({\text{C}}_{\text{GG}}/M_{\text{GG}}\;+\;{\text{C}}_{\text{Ge}}/M_{\text{Ge}})]$$

C_a_, C_GG_, and C_Ge_ represent the concentration of genipin, genipin-1-β-D-gentiobioside, and geniposide after enzymolysis of sulfatase, respectively. M_G_, M_GG-_, and M_Ge_ represent the mass of genipin, genipin-1-β-D-gentiobioside, and geniposide, respectively. The pharmacokinetic curves of phase II metabolites are illustrated in **Figure 4B**.

The pharmacokinetic behavior of the metabolites of rhein, emodin, and genipin in normal and cholestatic rat groups also differed significantly. In the cholestatic rats, the AUC_(0-∞)_ values of rhein and emodin decreased by 74.7 and 64.4%, respectively, compared with those of normal rats. A comparison of the Ka and Ke data indicated that the absorption and metabolism of the rhein and emodin phase II metabolites in the cholestatic rats was significantly inhibited, and this is similar to the metabolic behavior of the prototype drug. However, the AUC_(0-∞)_ and C_max_ values of genipin increased by 241.1 and 97.6%, respectively, compared with those of normal rats. Overall, a comparison of the Ka and Ke data indicated that the absorption and metabolism of the phase II genipin metabolite in cholestatic rats were stronger than those observed in normal rats. All the pharmacokinetic parameters of active constituents and phase II metabolites of YCHD in rats are listed in Supplementary Table S11.

### Ameliorative Effect on Rats With Cholestasis

#### Serum Biochemistry and Histopathological Examination Results

**FIGURE 5:**
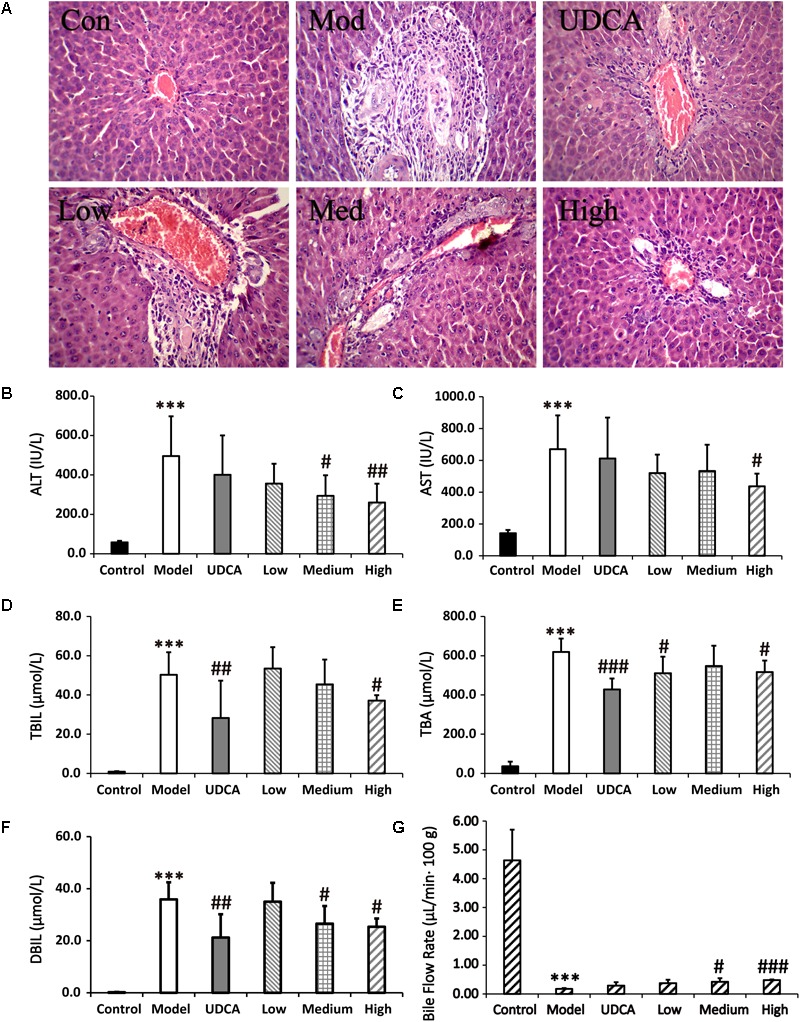
**(A)** Typical histopathological section photographs of rat liver speciments for H&E analysis (40x magnification). **(B–F)** The serum ALT, AST, TBIL, TBA, and DBIL levels, respectively. **(G)** Effect of bile flow rate over 360 min at 48 h after ANIT treatment. Control: vehicle only; Model: ANIT (50 mg/kg, i.g.); UDCA (100 mg/kg, i.g.) + ANIT (50 mg/kg, i.g.); Low: YCHD (6.0 g/kg, i.g.) + ANIT (50 mg/kg, i.g.); Medium: YCHD (9.0 g/kg, i.g.) + ANIT (50 mg/kg, i.g.); High: YCHD (12 gig, i.g.) + ANIT (50 mg/kg, i.g.); ANOVA test was used to calculate the significance of the differences, ^∗∗∗^*p* < 0.001 which compared with the control group; ^#^*p* < 0.05, ^##^*p* < 0.01, and ^###^*p* < 0.001 which compared with model group respectively.

The serum biochemistry results of ALT, AST, TBIL, TBA, and DBIL are presented in Supplementary Table S12 and **Figures 5B–F**. Serum ALT and AST are well-recognized markers of liver damage. As shown in **Figures 5B,C**, rats that were administered ANIT exhibited a remarkable increase in ALT and AST levels, compared with the control group, indicating that severe liver damage occurred after ANIT administration. The ALT levels were reduced in the rats that were treated with medium-dose (9 g/kg) YCHD; moreover, the ALT and AST levels were significantly reduced when the rats were treated with high-dose (12 g/kg) YCHD. Rats administered UDCA and low-dose (6 g/kg) YCHD exhibited a decrease in ALT and AST levels, although no statistical difference was observed. The TBIL, TBA, and DBIL levels, classical indicators of cholestatic liver injury, were markedly elevated in the ANIT-treated rats (**Figures 5D–F**), signifying that severe cholestasis had occurred. UDCA at a 50 mg/kg dose efficiently decreased the serum levels of TBIL, TBA, and DBIL to 43.8% (*p* < 0.01), 30.9% (*p* < 0.001), and 40.9% (*p* < 0.01), respectively, compared with the control group. Thus, UDCA had a moderate treatment effect on ANIT-induced severe cholestasis. The TBIL, TBA, and DBIL levels were significantly attenuated by 26.1, 16.6, and 29.4% (*p* < 0.05), respectively, in rats treated with YCHD at a dose of 12 g/kg (high dose), compared with the control group. Rats administered medium-dose (9 g/kg) YCHD showed reduced TBIL, TBA, and DBIL levels, although no statistically significant difference was observed. Overall, the results reveal that a high dose (12 g/kg) of YCHD had the best ameliorative effect on cholestasis, as exhibited in the significant improvement of ALT, AST, TBIL, DBIL, and TBA levels.

Blank, ANIT-treated, and UDCA- and YCHD-pretreated rat liver sections stained with hemotoxylin and eosin were examined by microscopy to provide visual evidence of the protective efficacy of YCHD on cholestasis. As shown in **Figure 5A**, the liver sections of the control rats (Con) showed normal hepatocyte structures. Marked cholestasis in terms of acute neutrophil infiltration, sinusoid congestion, and necrosis of the interlobular ducts and hepatocytes could be distinguished in specimens of ANIT-treated rats (Mod), compared with the normal liver morphologies in the blank control group. Concurrent administration of UDCA and 12.0 g/kg of YCHD resulted in hepatocyte hydropic degeneration with less neutrophil infiltration and a milder degree of bile duct epithelial damage, showing the greatest ameliorative effect. Specimens treated with 9.0 g/kg of YCHD displayed a slight reduction in inflammatory cell infiltration and other ANIT-induced histological damages. The observed liver damage in specimens treated with 6.0 g/kg of YCHD exhibited almost no attenuation of bile duct epithelial damage or portal tract edema. Therefore, we suggest that 12.0 g/kg of YCHD has clear beneficial effects in terms of biochemical indices and can significantly reduce liver damage in histological sections. In comparison, the efficacy of YCHD at doses of 6.0 and 9.0 g/kg is inferior to that of YCHD at 12.0 g/kg, thereby confirming the dose-related ameliorative effects of YCHD on cholestasis.

#### Effects of YCHD on Rat Bile Excretion

As a significant index in the ANIT-induced intrahepatic cholestasis, the bile flow rate was investigated in our experiments. **Figure 5G** shows that the bile was suppressed significantly (*p* < 0.001) in ANIT-induced cholestatic rat with the bile excretion of 0.18 ± 0.03 μL/min 100 g body weight (B.W.) in model group compared with normal group (4.64 ± 1.06 μL/min⋅100 g B.W.). After treatment of YCHD, the ANIT-suppressed bile flow was significantly (*p* < 0.05) increased to 0.42 ± 0.12 μL/min⋅100 g B.W. in medium-dose of YCHD group, and very significantly (*p* < 0.001) increased to 0.48 ± 0.02 μL/min⋅100 g B.W. in high-dose of YCHD group when compared with model group. Although the UDCA (0.29 ± 0.12 μL/min⋅100 g B.W.) and low-dose (0.38 ± 0.12 μL/min⋅100 g B.W.) group has no statistically significant increase in bile flow rate, there was a distinct increase in bile flow compared to the model group.

#### Metabolic Enzyme and Transporters Expression in Rat Liver

##### LC-MS/MS analysis

The expression levels of the metabolic enzyme UGT1A1 and the nine transporters (MRP2, BSEP, OCT1, NTCP, MATE1, MDR1, OATP1A1, OATP1A2, OATP1A4) were evaluated using the UHPLC-MS/MS method (**Table 3**). This direct quantitative method was used to quantify proteospecific peptides corresponding to specific proteins. All peptides were measured in positive ionization mode through ESI. The highest signal intensity of the protonated parent ions was observed, and the respective m/z ratios were applied as product ions.

**Table 3:** Multiple reaction monitoring parameters of peptides (and internal standard) selected for targeted analysis of hepatobiliary transporters.

Transporter	Signature peptides	Molecular weight	Parent ion (*z* = 2)	Product ion (*z* = 1)	Fragmentor	Collision energy (V)
Mrp2	LTIIPQDPILFSGSLR	1770.08	885.7	1329.9	200	25
Oct1	GVALPETIEEAENLGR	1697.84	849.7	1357.8	180	29
Ntcp	AAATEDATPAALEK	1358.42	680.0	915.5	140	18
	AAATEDATPAALE***K^∗^***	1366.42	684.0	923.0	140	23
Oatp1a4	TFQFPGDIESSK	1355.45	678.6	832.3	160	19
Mate1	LLLSGFQEELR	1304.48	652.9	965.0	140	24
Bsep	STALQLIQR	1029.19	515.5	529.5	130	17
Mdr1	NTTGALTTR	934	467.9	719.4	110	14
Oatp1a1	EENLGITK	903	452.3	468.1	120	8
Oatp1a2	SVQPELK	799.91	400.8	486.3	110	9
UGT1A1	SVFDQDPFLLR	1336.48	668.8	575.8	150	18

*^∗^Labeled amino acid residue of the internal standard.*

##### Method validation

The UHPLC-MS/MS method was found to be ideal for the determination of all 11 proteospecific peptides in the blank matrix EB II. The linearity, precision, accuracy, recovery, and stability of the method were validated. Calibration curves were prepared from 0.221 to 70.72 nM with a correlation coefficient (R^2^) of over 0.9901; the weighting factor was 1/x^2^ (in each case *n* = 8). The LLOQ of all quantitative assays ranged from 0.010 to 0.398 (S/N = 10) (Supplementary Table S13).

Intraday and interday accuracy and precision for all peptides in the validation matrix were within the range of ±15% of the nominal concentrations and <15% for the respective coefficients of variation of the mean values. The RSD value of the intraday and interday precision and accuracy was below 16.7% for all components at the three QC sample concentrations. Furthermore, the accuracy of the method was within the recommended range of acceptance, with the RE% ranging from -15.6 to 13.0% (Supplementary Table S14). The average recovery values of the QC samples were 100.1–149.1, 102.6–109.2, and 116.9–160.6% at low, medium, and high levels, respectively (Supplementary Table S15).

All peptides demonstrated sufficient stability (±20% of the initial concentrations at low, medium, and high concentrations) during storage for 24 h at room temperature (25°C) in the cooled autosampler rack and for 2 months at -20°C (Supplementary Table S16). Furthermore, the peptides were stable during three freeze–thaw cycles, as summarized in Supplementary Table S16. Overall, the obtained coefficients of variation for the stability of all analytes ranged from 82.9 to 116.8%, which is within an acceptable range; the chromatograms are presented in Supplementary Figure S6. All data with an acceptable range indicate that the peptides were stable under laboratory conditions and that the method is reliable for determination studies.

##### Method application

The developed method was applied to quantify the protein content of relevant metabolizing enzymes in rat liver microsomes.

**FIGURE 6:**
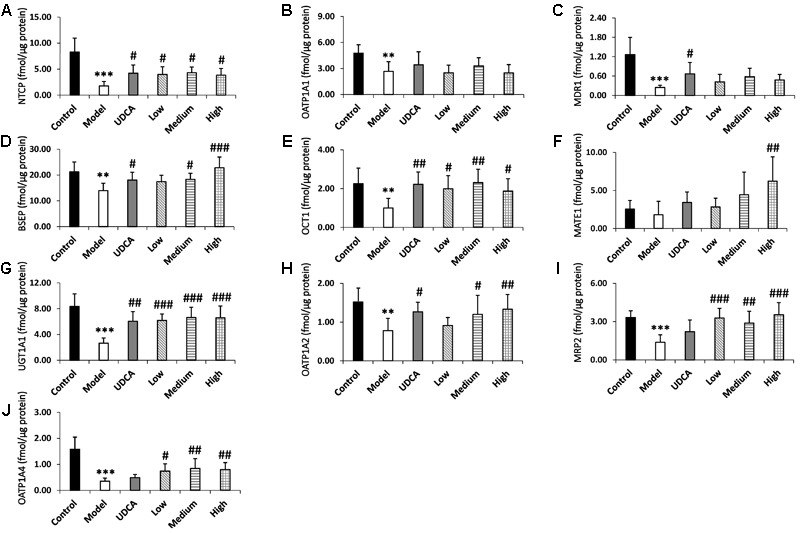
**(A–J)** The expression levels of metabolic enzyme UGT1A1 and nine transporters NTCP, OATP1A1, OATP1A2, OATP1A4, MDR1, BSEP, OCT1, MATE1, MRP2 in rat liver, respectively. ANOVA with the *post hoc* test was used to calculate the significance of the differences. ^∗∗^*p* < 0.01 and ^∗∗∗^*p* < 0.001 which compared with the control group; ^#^*p* < 0.05, ^##^*p* < 0.01, and ^###^*p* < 0.001 which compared with model group respectively.

The quantitative results of the metabolic enzyme UGT1A1 and the nine transporters MRP2, BSEP, OCT1, NTCP, MATE1, MDR1, OATP1A1, OATP1A2, and OATP1A4 are listed in Supplementary Table S17 and **Figure 6**. The downregulation of BSEP and MRP2 expression is a principal indicator of cholestasis (Orsler et al., 1999). The BSEP and MRP2 expression levels in our model group were significantly reduced by 37.1 and 57.0%, respectively, compared with the control group (**Figures 6D,I**), indicating that ANIT-induced cholestasis had been successfully established in this experiment. Furthermore, UGT1A1 and OCT1, NTCP, MDR1, OATP1A1, OATP1A2, and OATP1A4 of the model group decreased significantly by 68.1, 52.0, 76.7, 79.1, 40.8, 44.4, and 75.1% (*p* < 0.01), respectively, compared with the control group. UDCA showed adequate ameliorative effects on cholestasis; the treated rats manifested a 2.1-fold higher level of UGT1A1 (*p* < 0.01), as well as 2.2-, 2.4-, 1.2-, 2.1-, and 1.5-fold higher levels of NTCP, MDR1, BSEP, OCT1, and OATP1A2 (*p* < 0.05), respectively, compared with the control group. UDCA also upregulated the expression of MATE1 (1.7-fold), MRP2 (1.5-fold), and OATP1A4 (1.3-fold); however, these were not statistically significant. YCHD exhibited ameliorative effects on cholestasis, mainly in the upregulation of UGT1A1 and MRP2 (**Figure 6G**, *p* < 0.001; I, *p* < 0.01). Furthermore, a high dose of YCHD had the greatest ameliorative effect on cholestasis, increasing the UGT1A1, MRP2, BSEP levels by 2.4-, 2.4-, and 1.6-fold, respectively (**Figures 6D,G,I**, *p* < 0.001); OATP1A2 and OATP1A4 levels by 1.6- and 1.3-fold, respectively (**Figures 6H,J**, *p* < 0.01); and OCT1and NTCP levels 1.8- and 2.0-fold, respectively (**Figures 6A,E**, *p* < 0.05). In addition to the high dose, both low and medium doses of YCHD still exhibited a limited ameliorative effect. In the livers of the medium-dose group, the UGT1A1, OCT1, MRP2, OATP1A4, NTCP, BSEP, and OATP1A2 levels were significantly increased by 2.4- (*p* < 0.001), 2.1-, 1.9-, and 2.2- (*p* < 0.01), 2.3-, 1.3-, and 1.4-fold (*p* < 0.05), respectively, compared with the control group. The low dose of YCHD also increased the expression of UGT1A1 and MRP2 by 2.2-fold (**Figures 6G,I**, *p* < 0.001), in addition to increasing the expression of NTCP, OCT1, and OATP1A4 by 2.1-, 1.9-, and 2.0-fold (**Figures 6A,E,J**, *p* < 0.05), respectively. The results shows that ANIT induced cholestasis is associated with down-regulation of UGT1A1, MRP2, BSEP, OCT1, NTCP, MDR1, OATP1A1, OATP1A2, and OATP1A4 and YCHD can up-regulate UGT1A1, MRP2, BSEP, OATP1A2, OATP1A4, OCT1, and NTCP during the ameliorative effect of cholestasis.

## Discussion and Conclusion

The metabolic process of bilirubin principally consists of bilirubin liver cell uptake, transportation, combined reaction, and excretion (Sticova and Jirsa, 2013). In blood, the unbound unconjugated bilirubin (UCB) is rapidly taken up by hepatocytes under the mediation of the organic anion-transporting peptides (OATPs) 1B1 and 1B3, located in the lateral basement of a liver cell (Hoekstra et al., 2013). In the normal physiological state, fat-soluble UCB is converted to water-soluble conjugated bilirubin (CB) under the selective catalysis of UDP-glucuronosyltransferase 1-1 (UGT1A1), and the partial or complete loss of UGT1A1 activity has been reported to cause disordered accumulation of UCB in plasma as well as increased levels of bilirubin in the blood. Thus, the UGT1A1-mediated glucuronidation process is a crucial step in promoting the elimination of potentially toxic bilirubin (Kadakol et al., 2000). CB can be transported through multidrug resistance-associated protein 2 (MRP2) on the capillary bile duct membrane of hepatocytes into the capillary bile duct, discharged into the small intestine with bile, and excreted through the intestine. In the metabolism of bilirubin, the bile salt export pump (BSEP) is a crucial protein that regulates bile production and maintains normal functioning of the liver. BSEP inhibition or downregulation can lead to bile retention or intrahepatic cholestasis. Furthermore, there are also many other transporters involved in drugs and endogenous substances absorption and metabolism. For example, multidrug resistance protein 1 (MDR1), also termed P-glycoprotein or *ABCB1* gene, the physiological function of this protein lies in protecting the whole body against a broad variety of toxic compounds such as digoxin and paclitaxel (Borst and Schinkel, 2013). Uptake of compounds into the hepatocyte is mediated by the sodium-dependent taurocholate cotransporter (NTCP) and sodium-independent organic anion transporters belonging to the OATP family, of which three members are present in the basolateral membrane. Several transporters of the SLC22 family of solute carriers were also present in the basolateral membrane of hepatocytes, such as organic cation transporter 1 (OCT1) (Koepsell, 2013). Multidrug and toxin extrusion protein 1 (MATE1) is a drug transporter located in the brush-border membrane and mediates the export of drugs such as metformin from the tubule cell into the tubular lumen (Muller and Fromm, 2011; Nies et al., 2011; Konig et al., 2013; Motohashi and Inui, 2013). OATPs are transmembrane transporters that primarily mediate the cellular uptake of a broad range of exogenous compounds and organic endogenous (Stieger and Hagenbuch, 2014). For example, OATP1A1, A2, and A4 mediate sodium-independent transport in a wide range of amphipathic organic compounds including bile salts, anionic oligopeptides, steroid conjugates, as well as numerous drugs, and other xenobiotic substances (Hata et al., 2003). Overall, monitoring the changes in the expression of these transporters is more conducive to revealing the mechanism of YCHD’s treatment effect on ANIT-induced cholestasis.

Alpha-naphthylisothiocyanate, which can cause an intrahepatic cholestasis in rodents, is commonly used to create human intrahepatic cholestasis models (Cui et al., 2009) and conduct both *in vitro* and *in vivo* toxicological research. And it is a classic hepatotoxic agent that induces cholestasis by injuring biliary epithelial cells and hepatocytes (Plaa and Priestly, 1976) which characterized by bile duct obstruction, severe interlobular duct epithelial apoptosis or necrosis, and neutrophil infiltration around bile ducts (Kodali et al., 2006), these condition are morphologically similar to drug-induced cholangitic cholestasis in humans (Ungar and Popp, 1976). Several studies have shown that significant cholestasis occurs after treatment with ANIT for 24–48 h (Connolly et al., 1988). Emerging evidence suggests that bile duct epithelial cells are primary targets of ANIT under cholestatic conditions. Unstable glutathione-conjugated ANIT is transported by MRP2 into the biliary canalicular, where free ANIT is then dissociated and acutely or chronically impairs the bile duct (Orsler et al., 1999). And a recent study reported that ANIT induced intracellular bile acid accumulation through the adenosine monophosphate-activated protein kinase-farnesyl X receptor (AMPK-FXR) pathways, and this accumulation was accompanied by a significant reduced in the expression of bile acid transporters like OATP2, MRP2, MRP3, BSEP et al. (Luyendyk et al., 2011; Li et al., 2016), subsequently inducing intrahepatic cholestasis. ANIT was also reported to directly suppress MRP2, MRP3, NTCP, and OATP2 in rat livers (Luyendyk et al., 2011; Guo et al., 2014). The inhibition of BSEP by ANIT was proven (Chen et al., 2016). Downregulation of these transporters inhibits the secretion of bile salts. Therefore, ANIT-induced cholestasis model has clinical significance for understanding the pathogenesis and potential therapeutic targets of drug-induced cholestasis in humans. Generally, increased blood ALT and AST levels are used as diagnostic indicators for hepatocyte damage, while serum TBIL, DBIL, and TBA are commonly considered as the biomarkers of cholestasis (Zhu et al., 2018). In the present study, cholestatic rat model was successfully established that rats exposed to ANIT (50 mg/kg i.g.) had significantly increased serum levels of ALT, AST, TBIL, DBIL and TBA, combined with the suppressed bile flow rate, and significantly inhibition of expression levels of UGT1A1, OATP1A4, MRP2, NTCP, OCT1, and OATP1A2 by 65.1, 75.1, 57.0, 79.1, 76.7, 44.4%, respectively, in cholestasis rats. And these changes may account for reducing the uptake of bilirubin and metabolic process of YCHD in cholestatic rat liver.

Instead of focusing on a single target, a paradigm shift has taken place that puts new interest in agents that regulate multiple targets simultaneously with less side effects and lower toxicity. Combinations with multiple effector pathways and multiple targets are often more effective than single agents and may help address many of the treatment-related challenges. However, if we are to develop effective, customized multidrug programs, continued progress will be crucial. The efficacy of traditional Chinese medicine formula based on the combined effect of the mixture of ingredients provides new therapeutic opportunities. YCHD is a typical example, and its efficacy in treating cholestasis has been widely reported (Chen et al., 2015; Yan et al., 2017). In our research, a high dose (12 g/kg) of YCHD exhibits a greatest ameliorative effect on ANIT-induced cholestasis, particularly in reducing ALT, AST, TBIL, DBIL, and TBA levels by 47.6, 34.9, 26.1, 29.4, and 16.6%, respectively, and also improving the pathological state of hepatocytes. Increased expression of UGT1A1, BSEP, MRP2, OATP1A4 by 136.5, 58.3, 137.8, and 115.9% in cholestasis rats after YCHD treatment reflects that YCHD may promote the metabolism of bilirubin through up-regulation of levels of metabolic enzymes and transporters in rat liver. In particular, UGT1A1 specifically catalyzes the phase II metabolic binding reaction in hepatocytes, and the transport protein MRP2 is a multi-specific organic anion transporter that mediates the efflux of glucorinidated/sulfated bile acids or bilirubin (Kullak-Ublick et al., 2000). Hepatobiliary transport of bile acids are regulated by many special transporters, inhibited or expressed in the canalicular membrane of the hepatocyte (Arab et al., 2009). Bsep and Mrp2 are critically involved in this process, and many kinds of bile acid transporters have been demonstrated to play a vital role to maintain hepatic bile acid homeostasis in absorption and excretion (Yang et al., 2009). The regulation of YCHD on liver metabolic enzymes and transporter, which were closely correlation with bilirubin metabolism, in present study would make its mechanism clearer.

By using UHPLC/Q-TOF-MS, 11 original active components and 8 phase II metabolites have been detected in rats after oral administration of YCHD, including rhein-11-*O*-gluA, genipin-10-*O*-gluA, genipin-10-*O*-SO_3_, caffeic acid-1-*O*-SO_3_, caffeic acid-1-*O*-gluA, 4-hydroxyacetophenone-1-*O*-SO_3_, emodin-3-*O*-SO_3_ and emodin-3-*O*-gluA. Particularly, caffeic acid-1-*O*-SO_3_, caffeic acid-1-*O*-gluA, and 4-hydroxyacetophenone-1-*O*-SO_3_ are first detected after oral administration of YCHD. Furtherly, pharmacokinetic characteristics of eight components and four phase II metabolites are investigated by an efficient UHPLC-MS/MS method in cholestasis rats and normal rats. It is the most comprehensive study up to now, which could offer more informations than previous references. In pharmacokinetic study of YCHD, 65.9 and 73.4% rhein exist in normal and cholestatic rats in the form of the phase II metabolites. Prototype of capillarisin, emodin, and genipin can’t be detected neither in normal nor cholestatic rats before hydrolysis, which shows that these three components mainly present in glucuronic acid and sulfuric acid conjugation. All the Tmax of rhein, 4-hydroxyacetophenone, genipin-1-β-D-gentiobiodide, caffeic acid, chlorogenic acid and geniposide, phase II metabolites of emodin and capilarisin in normal and cholestatic rats after oral administration of YCHD are within 27 min, which suggests these components are absorbed rapidly. And the mean Tmax values of detected eight prototypes and phase II metabolites of genipin and emodin are smaller in the normal group than that in the cholestasis group, also the smaller mean Ka values of rhein, 4-hydroxyacetophenone, genipin-1-β-D-gentiobiodide, scandoside methyl ester, caffeic acid, chlorogenic acid and phase II metabolites of rhein, emodin in model group implies the pathological state of cholestasis can hinder the absorption of these compounds. Nevertheless, the smaller Tmax value of phase II metabolites of rhein in cholestatic rats means the absorption of it can be accelerated in cholestasis rats. In contrary, the Tmax values for geniposidic acid are obviously larger than other components, which means it is more difficult to be absorbed into blood. The most interesting discovery is the distinctive pharmacokinetic characteristics of geniposidic acid, geniposide and phase II metabolites of genipin, which can be clearly observed from the drug time curve. The AUC(0-∞) and Cmax of these components are larger in cholestasis rats than that in normal rats, while pharmacokinetics of other compounds perform the opposite in both groups. And it requires further experiments to explain this phenomenon.

**FIGURE 7:**
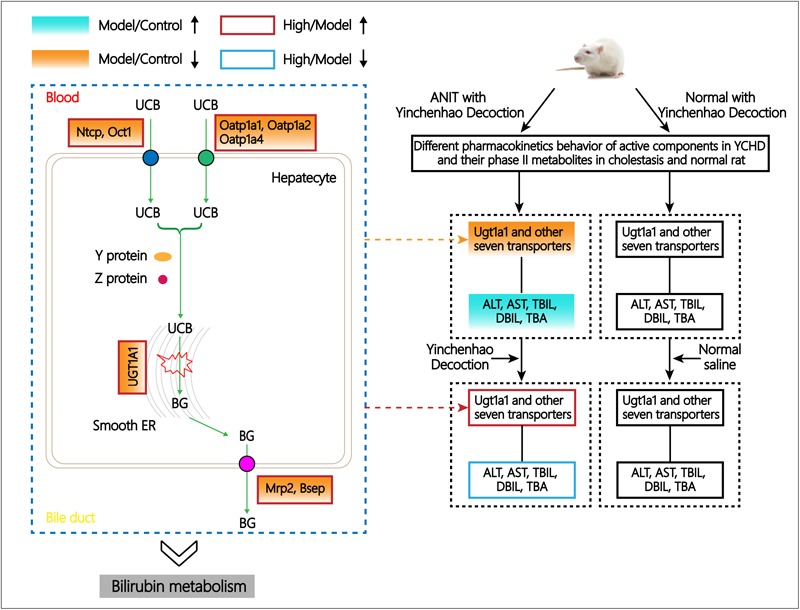
Schematic diagram of the regulative metabolic pathway of bilirubin related to ANIT and YCHD treatment.

As summarized in **Figure 7**, pharmacokinetic characteristics of the eight prototype components of YCHD and their phase II metabolites in normal and cholestatic model rats were distinctly different that most of the active constituents and phase II metabolites of YCHD were inhibited in cholestatic rats, suggesting that the absorption and excretion of YCHD may be affected by pathological state, thus it can induce different pharmacological effects and which is probable the key of treating mechanism. Indeed, the expression levels of the metabolic enzyme UGT1A1 and eight transporters including MRP2, BSEP, OCT1, NTCP, MDR1, OATP1A1, OATP1A2, and OATP1A4, were all down-regulated significantly in cholestatic model rats when compared with normal rats. The results indicate that UGT1A1, MRP2, BSEP, OCT1, NTCP, OATP1A2, and OATP1A4 can be used as potential biological markers for evaluating the ameliorative effects of YCHD in cholestasis, and considering that the metabolism of drugs and endogenous substances (like bilirubin, bile acid, and so on) are related to metabolic enzyme of UGT1A1 and transporter protein of OATP1B1, MRP2 in liver (Cui et al., 2009; Vitek and Ostrow, 2009; Goto and Takikawa, 2010), these enzymes directly affect the metabolic process of YCHD and bilirubin. Moreover, different doses of YCHD can have different effects on cholestasis, which can also be reflected by decreased levels of serum biochemistry. Thus, it is hoped that our findings will provide a novel perspective on the mechanism of YCHD in treating cholestasis.

## Ethics Statement

All care and handling of the animals were performed in accordance with the requirements of the Institutional Animal Care and Use Committee of Shanghai University of Traditional Chinese Medicine. All experimental protocols have been reviewed and approved by the Institutional Animal Experimental Ethics Committee of Shanghai University of Traditional Chinese Medicine.

## Author Contributions

TZ and YD formulated the study concept and design of this paper, guided the critical revision of the manuscript and provide important intellectual content. Y-XY performed the research, acquired and analyzed the data, drafted the manuscript. YZ and PK helped in executing part of research. Z-ZC, FS, and N-HM improved and approved the manuscript. All the authors have reviewed the manuscript, agreed to all the contents and agreed the submission.

## Conflict of Interest Statement

The authors declare that the research was conducted in the absence of any commercial or financial relationships that could be construed as a potential conflict of interest.
