# Supplementary material for: Yinchenhao Decoction Ameliorates Alpha-Naphthylisothiocyanate Induced Intrahepatic Cholestasis in Rats by Regulating Phase II Metabolic Enzymes and Transporters
Source: Front Pharmacol. 2018 May 15;9:510. doi: 10.3389/fphar.2018.00510 (PMC5962729; doi:10.3389/fphar.2018.00510)
Supplement: Supplementary file 1 [file Data_Sheet_1.docx]

***Supplementary materials for*：**

**Yinchenhao Decoction Ameliorates Alpha-naphthylisothiocyanate Induced Intrahepatic Cholestasis in Rats by Regulating Phase II Metabolic Enzymes and Transporters**

Ya-Xiong Yi^1^, Yue Ding^2*^, Yong Zhang^2^, Ning-Hui Ma^1^, Feng Shi^3^, Ping Kang^4^, Zhen-Zhen Cai^5^, Tong Zhang^1*^

^1^ School of Pharmacy, Shanghai University of Traditional Chinese Medicine, Shanghai, China

^2^ Experiment Center for Teaching and Learning, Shanghai University of Traditional Chinese Medicine, Shanghai, China

^3^ Pharmaceutical Preparation Section, Guangming Chinese Medicine Hospital of Pudong New Area, Shanghai, China

^4^ [Headmaster's office](http://www.baidu.com/link?url=5i2fM8fDGmABLl-EcYNYQG-bXdNXJsQDq2ElJpl3p1nHBfXxnyjlMn3G9uxoM35z6L3lnWxh3wKJhg72C_lDiyp4EFnzJ0_jfRvIYw7pZkLTbfk2pKQadZlXSCDDzvPv), Shanghai University of Traditional Chinese Medicine, Shanghai, China

^5^ Experiment Center for Science and Technology, Shanghai University of Traditional Chinese Medicine, Shanghai, China

*** Correspondence:**

**Corresponding Author**

Yue Ding, Experiment Center for Teaching and Learning, Shanghai University of Traditional Chinese Medicine,1200 Cailun Road, Shanghai, 201203, China. Tel.: +86 021 51322325, E-mail address: dingyue-2001@hotmail.com.

Tong Zhang, Experiment Center for Teaching and Learning, Shanghai University of Traditional Chinese Medicine, 1200 Cailun Road, Shanghai, 201203, China. Tel.: +86 021 51322318, E-mail address: zhangtdmj@hotmail.com.

**1. Identification of Phase II Metabolites of Yinchenhao Decoction in Rat by UHPLC/Q-TOF-MS**

Separations were conducted on an Agilent SB-C18 column (2.1 mm×50 mm, 1.8 μm) of a UHPLC/QTOF-MS system (Waters Corp., Milford, MA, USA) maintained at 40 ℃. The column was eluted with 0.4 % formic acid in water (A) and acetonitrile (B) as mobile phase under the following gradient conditions: 0-1 min, 5%-5 % B; 1-4 min, 5%-60 % B, 4-5 min, 60%-70 % B 5-5.1 min, 70%-5% B, and re-equilibration for 2 min with 2% B. The flow rate was set at 0.4 mL/min, and the injection volume was 5 μL, respectively. Figure S1 shows the HPLC profile of phase II metaYinchenhao Decoction (YCHD). M1~8 stand for rhein-11-O-gluA, genipin-10-O-gluA, genipin-10-O-SO_3_, caffeic acid-1-O- SO_3_, caffeic acid-1-O-gluA, 4-hydroxyacetophenone-1-O-SO_3_, emodin-3-O-SO_3_, emodin-3-O-gluA, respectively.


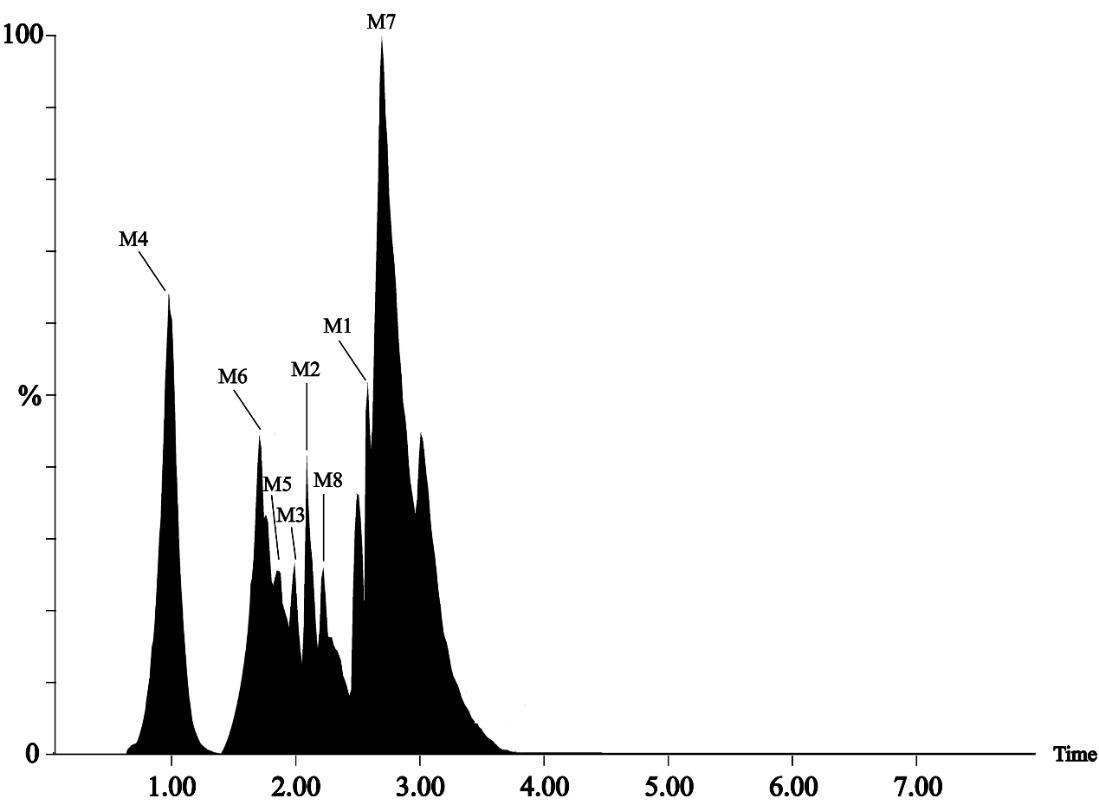


**Figure S1.** Identification chromatogram of eight phase II metabolites of Yinchenhao Decoction in rat by UHPLC/Q-TOF-MS

**2. Pharmacokinetics Study on the Main Components of the Yinchenhao Decoction and Its Part of the II Phase Metabolites by UHPLC-MS/MS**

**2.1 Method Validation of ten components in prior-enzymolysis sample**

The method validation of ten components in samples without being hydrolyzed by sulfatase was conducted as described in section 2.4.3. Chromatograms are listed in Figure S2 and S3 and the summarized data can be seen in Table S1-S5.


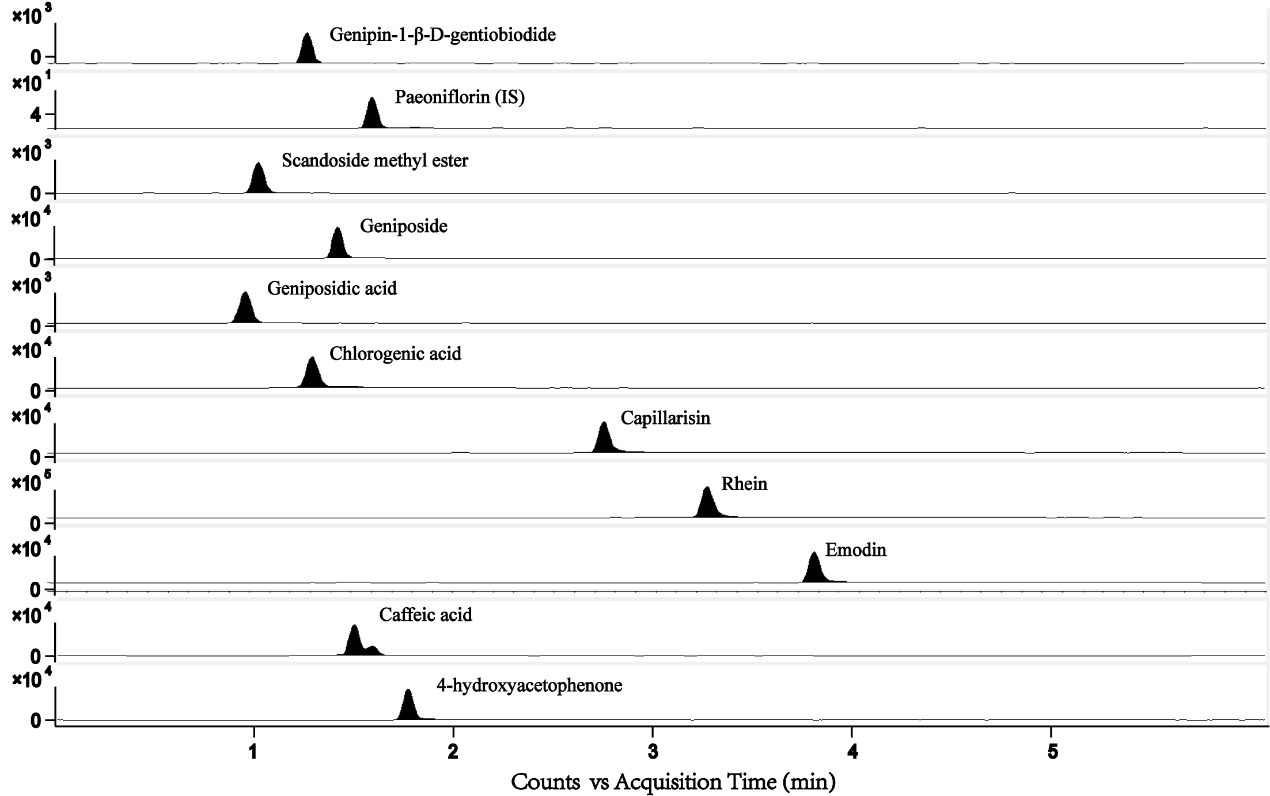


**Figure S2** Multiple chromatograms of ten analytes and internal standard.


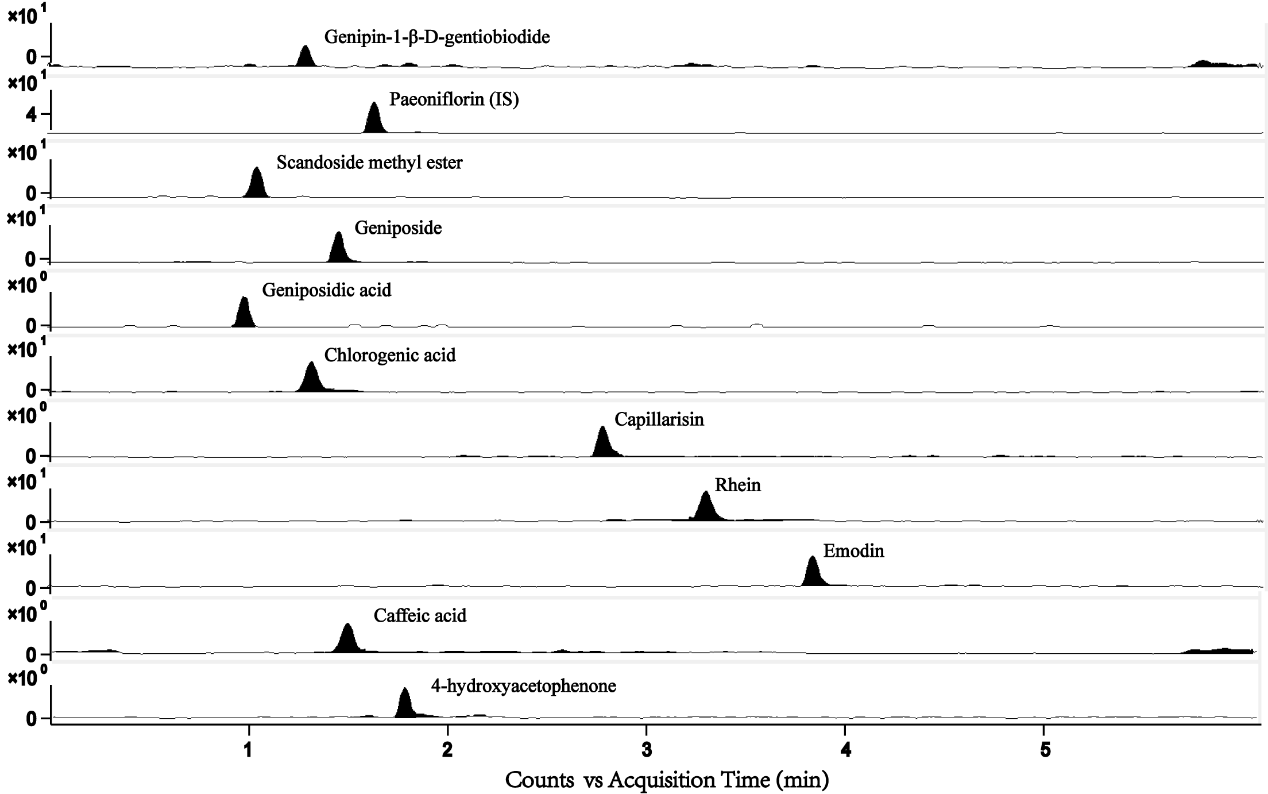


**Figure S3** Multiple chromatograms of ten components and internal standard in plasma.

**TABLE S1** The Linearity Data, Calibration Range and LLOQ of Ten Compounds.

| Component | Calibration Range  (ng/mL) | R^2^ | Calibration Range (ng/mL) | LLOQ  (ng/mL) |
| --- | --- | --- | --- | --- |
| Genipin-1-β-D-gentiobiodide | Y=0.003093*X+0.017505 | 0.9812 | 10.00-640.0 | 5.43 |
| Scandoside methyl ester | Y=0.005781*X+0.027630 | 0.9926 | 10.94-700.0 | 7.20 |
| Geniposide | Y=9.301334*10^-4^X+0.007394 | 0.9911 | 12.97-1660 | 1.38 |
| Geniposidic acid | Y=0.004147*X+0.031451 | 0.9900 | 12.19-3120 | 0.21 |
| Chlorogenic | Y=0.008064*X+0.024517 | 0.9910 | 11.80-755.0 | 1.20 |
| Capillarisin | Y=0.017420*X-0.055301 | 0.9933 | 8.59-550.0 | 0.52 |
| Rhein | Y=0.020218*X+0.081065 | 0.9948 | 5.07-5840 | 2.26 |
| Emodin | Y=0.009305*X+0.029165 | 0.9935 | 7.07-905.0 | 0.64 |
| Caffeic acid | Y=0.019277*X+0.181859 | 0.9919 | 5.66-725.0 | 1.56 |
| 4-hydroxyacetophenone | Y=0.005068*X+0.031478 | 0.9915 | 10.39-2660 | 3.88 |

**TABLE S2** Intra- and Inter-day Precision and Accuracy of the Assay

| Intra-Day (n=5) | | | | | | | | | | |
| --- | --- | --- | --- | --- | --- | --- | --- | --- | --- | --- |
|  | Added | Founded | RSD | Accuracy |  | Added | Founded | RSD | Accuracy |  |
|  | (ng/mL) | (ng/mL) | (%) | (RE %) |  | (ng/mL) | (ng/mL) | (%) | (RE %) |  |
| Em | 7.07 | 7.62±0.87 | 11.4 | 7.80 | SME | 10.94 | 10.10±1.17 | 11.6 | -7.65 |  |
|  | 113.1 | 111.5±9.72 | 8.7 | -1.45 |  | 87.50 | 90.39±12.78 | 14.1 | 3.30 |  |
|  | 905.0 | 1052±39.55 | 3.8 | 16.25 |  | 700.0 | 746.2±26.69 | 3.6 | 6.59 |  |
| Rh | 5.70 | 5.82±0.53 | 9.1 | 2.08 | CaA | 5.66 | 5.11±0.86 | 16.8 | -9.76 |  |
|  | 91.25 | 88.62±4.78 | 5.4 | -2.88 |  | 90.63 | 93.79±2.40 | 2.6 | 3.49 |  |
|  | 730.0 | 810.7±52.56 | 6.5 | 11.05 |  | 725.0 | 787.7±34.00 | 4.3 | 8.65 |  |
| 4-Hy | 10.39 | 10.31±0.80 | 7.8 | -0.76 | ChA | 11.80 | 11.47±0.93 | 8.1 | -2.78 |  |
|  | 83.13 | 83.05±7.30 | 8.8 | -0.09 |  | 94.38 | 95.12±4.91 | 5.2 | 0.79 |  |
|  | 665.0 | 664.6±27.73 | 4.2 | -0.06 |  | 755.0 | 851.6±26.44 | 3.1 | 12.79 |  |
| GA | 12.97 | 13.89±1.31 | 9.5 | 7.09 | Cap | 4.30 | 4.81±0.46 | 9.5 | 11.94 |  |
|  | 103.8 | 118.7±13.86 | 11.7 | 14.42 |  | 68.75 | 72.40±3.01 | 4.2 | 5.31 |  |
|  | 830.0 | 866.9±49.78 | 5.7 | 4.44 |  | 550.0 | 613.6±34.28 | 5.6 | 11.56 |  |
| GG | 10.00 | 10.18±1.14 | 11.2 | 1.81 | Ge | 12.19 | 12.92±1.37 | 10.6 | 6.03 |  |
|  | 80.00 | 72.54±7.57 | 10.4 | -9.33 |  | 97.50 | 98.76±10.81 | 10.9 | 1.29 |  |
|  | 640.0 | 682.1±84.20 | 12.3 | 6.58 |  | 780.0 | 828.5±30.35 | 3.7 | 6.22 |  |

| Inter-Day (n=5 series per day, for 3 days) | | | | | | | | | |
| --- | --- | --- | --- | --- | --- | --- | --- | --- | --- |
|  | Added | Founded | RSD | Accuracy |  | Added | Founded | RSD | Accuracy |
|  | (ng/mL) | (ng/mL) | (%) | (RE %) |  | (ng/mL) | (ng/mL) | (%) | (RE %) |
| Em | 7.07 | 7.60±0.64 | 8.5 | 7.43 | SME | 10.94 | 11.37±0.30 | 2.6 | 3.91 |
|  | 113.1 | 115.3±6.64 | 5.8 | 1.94 |  | 87.50 | 84.46±10.27 | 12.2 | -3.47 |
|  | 905.0 | 1023±151.5 | 14.8 | 12.99 |  | 700.0 | 765.9±76.48 | 10.0 | 9.41 |
| Rh | 5.70 | 6.18±0.34 | 5.5 | 8.39 | CaA | 5.66 | 6.27±1.05 | 16.7 | 10.66 |
|  | 91.25 | 91.25±4.55 | 5.0 | 0.00 |  | 90.63 | 91.64±14.88 | 16.2 | 1.13 |
|  | 730.0 | 808.8±41.02 | 5.1 | 10.80 |  | 725.0 | 785.8±73.42 | 9.3 | 8.39 |
| 4-Hy | 10.39 | 10.37±0.06 | 0.6 | -0.17 | Ch | 11.80 | 10.54±1.35 | 12.8 | -10.64 |
|  | 83.13 | 79.01±3.51 | 4.4 | -4.95 |  | 94.38 | 106.0±9.43 | 8.9 | 12.32 |
|  | 665.0 | 710.6±54.50 | 7.7 | 6.85 |  | 755.0 | 870.4±16.38 | 1.9 | 15.28 |
| GA | 12.97 | 14.18±0.29 | 2.0 | 9.36 | Cap | 4.30 | 8.30±0.76 | 9.2 | -3.40 |
|  | 103.8 | 111.2±10.30 | 9.3 | 7.22 |  | 68.75 | 70.10±6.82 | 9.7 | 1.96 |
|  | 830.0 | 900.1±81.80 | 9.1 | 8.45 |  | 550.0 | 620.6±32.94 | 5.3 | 12.84 |
| GG | 10.00 | 10.26±0.42 | 4.1 | 2.62 | Ge | 12.19 | 11.92±1.34 | 11.3 | -2.21 |
|  | 80.00 | 75.03±4.32 | 5.8 | -6.21 |  | 97.50 | 100.1±10.04 | 10.0 | 2.62 |
|  | 640.0 | 676.3±30.06 | 4.4 | 5.67 |  | 780.0 | 848.0±68.14 | 8.0 | 8.72 |

GA, Geniposidic acid; SME, Scandoside methyl ester; Cap, Capillarisin; Ch, Chlorogenic acid; GG, Genipin-1-β-D–gentiobioside; CaA, caffeic acid; Ge, Geniposide; 4-Hy, 4-hydroxyacetophenone; Rh, Rhein; Em, Emodin;

**TABLE S3** Matrix Effects for the Detection of Ten Compounds (n = 5).

|  | Concentration | ME | RSD |  | Concentration | ME | RSD |
| --- | --- | --- | --- | --- | --- | --- | --- |
|  | (ng/mL) | (%) | (%) |  | (ng/mL) | (%) | (%) |
| Em | 7.07 | 101.2 | 1.7 | SME | 10.94 | 53.8 | 6.9 |
|  | 113.1 | 114.4 | 6.3 |  | 87.50 | 44.8 | 7.8 |
|  | 905.0 | 116.2 | 1.9 |  | 700.0 | 52.0 | 12.2 |
| Rh | 5.70 | 95.2 | 1.3 | CaA | 5.66 | 101.9 | 5.1 |
|  | 91.25 | 94.4 | 2.2 |  | 90.63 | 92.6 | 2.1 |
|  | 730.0 | 95.3 | 1.7 |  | 725.0 | 97.2 | 3.0 |
| 4-Hy | 10.39 | 84.6 | 1.7 | Ch | 11.80 | 102.9 | 4.5 |
|  | 83.13 | 82.3 | 6.4 |  | 94.38 | 92.2 | 1.2 |
|  | 665.0 | 86.3 | 3.0 |  | 755.0 | 102.6 | 1.5 |
| GA | 12.97 | 66.6 | 5.1 | Cap | 4.30 | 96.3 | 8.2 |
|  | 103.8 | 65.7 | 4.0 |  | 68.75 | 106.9 | 3.7 |
|  | 830.0 | 75.0 | 6.4 |  | 550.0 | 95.4 | 1.1 |
| GG | 10.00 | 70.4 | 15.6 | Ge | 12.19 | 66.2 | 4.4 |
|  | 80.00 | 65.2 | 8.8 |  | 97.50 | 63.8 | 6.9 |
|  | 640.0 | 64.0 | 7.1 |  | 780.0 | 65.6 | 7.8 |

**TABLE S4** Recoveries for the Detection of Ten Compounds (n = 5).

|  | Concentration | RE | RSD |  | Concentration | RE | RSD |
| --- | --- | --- | --- | --- | --- | --- | --- |
|  | (ng/mL) | (%) | (%) |  | (ng/mL) | (%) | (%) |
| Em | 7.07 | 50.4 | 3.9 | SME | 10.94 | 48.1 | 17.0 |
|  | 113.1 | 66.7 | 2.4 |  | 87.50 | 47.4 | 8.0 |
|  | 905.0 | 81.5 | 1.5 |  | 700.0 | 50.0 | 4.2 |
| Rh | 5.70 | 70.0 | 2.8 | CaA | 5.66 | 101.9 | 5.1 |
|  | 91.25 | 68.3 | 1.3 |  | 90.63 | 75.1 | 4.1 |
|  | 730.0 | 75.5 | 0.6 |  | 725.0 | 94.5 | 2.4 |
| 4-Hy | 10.39 | 67.3 | 2.7 | Ch | 11.80 | 98.8 | 2.1 |
|  | 83.13 | 62.6 | 3.8 |  | 94.38 | 90.3 | 2.7 |
|  | 665.0 | 75.5 | 1.4 |  | 755.0 | 95.2 | 0.7 |
| GA | 12.97 | 68.4 | 11.0 | Cap | 4.30 | 82.8 | 13.0 |
|  | 103.8 | 73.7 | 2.6 |  | 68.75 | 76.3 | 2.9 |
|  | 830.0 | 76.7 | 2.8 |  | 550.0 | 79.2 | 3.0 |
| GG | 10.00 | 62.8 | 3.4 | Ge | 12.19 | 53.4 | 10.3 |
|  | 80.00 | 61.6 | 4.9 |  | 97.50 | 62.4 | 6.2 |
|  | 640.0 | 64.6 | 1.5 |  | 780.0 | 66.8 | 6.3 |

**TABLE S5** Stability of the Ten Compounds.

|  |  | Accuracy (RE %) | | | |
| --- | --- | --- | --- | --- | --- |
| Compound | Concentration (ng/mL) | Post-Preparative  4℃,for 24 h | Short-Term  25℃for 24 h | Freezing-Thawing  -70℃, 3 cycles | Long-Term  -70℃, 2 months |
| Em | 7.07 | 92.43 | 99.9 | 98.0 | 112.0 |
|  | 113.1 | 86.69 | 98.4 | 92.6 | 92.1 |
|  | 905.0 | 105.4 | 100.3 | 99.6 | 92.1 |
| Rh | 5.70 | 100.6 | 91.0 | 106.4 | 109.8 |
|  | 91.25 | 87.79 | 82.7 | 86.0 | 105.6 |
|  | 730.0 | 106.0 | 94.2 | 111.8 | 105.6 |
| 4-Hy | 10.39 | 94.32 | 88.2 | 106.4 | 86.0 |
|  | 83.13 | 105.4 | 86.4 | 111.0 | 107.6 |
|  | 665.0 | 95.02 | 97.2 | 113.2 | 115.3 |
| GA | 12.97 | 71.57 | 89.8 | 97.4 | 112.1 |
|  | 103.8 | 101.4 | 95.8 | 94.0 | 109.9 |
|  | 830.0 | 108.0 | 100.4 | 102.5 | 104.6 |
| GG | 10.00 | 107.5 | 116.0 | 97.7 | 85.0 |
|  | 80.00 | 86.42 | 93.4 | 100.6 | 103.7 |
|  | 640.0 | 84.30 | 97.0 | 100.5 | 102.5 |
| SME | 10.94 | 91.48 | 105.6 | 85.1 | 86.7 |
|  | 87.50 | 94.54 | 83.3 | 104.6 | 99.0 |
|  | 700.0 | 95.21 | 89.5 | 100.4 | 97.1 |
| CaA | 5.66 | 85.6 | 109.0 | 129.2 | 101.7 |
|  | 90.63 | 109.6 | 90.1 | 88.0 | 94.9 |
|  | 725.0 | 115.4 | 100.1 | 102.6 | 92.2 |
| Ch | 11.80 | 98.91 | 100.5 | 106.2 | 96.6 |
|  | 94.38 | 101.6 | 113.1 | 90.5 | 98.4 |
|  | 755.0 | 108.7 | 88.0 | 103.7 | 96.8 |
| Cap | 4.30 | 98.18 | 101.5 | 101.3 | 110.9 |
|  | 68.75 | 99.64 | 84.6 | 87.8 | 103.0 |
|  | 550.0 | 113.0 | 98.0 | 112.5 | 114.7 |
| Ge | 12.19 | 72.09 | 101.4 | 114.6 | 94.0 |
|  | 97.50 | 85.70 | 93.4 | 89.4 | 109.8 |
|  | 780.0 | 102.1 | 104.8 | 100.4 | 104.5 |

**2.2 Method Validation of four components in** **post-enzymolysis sample**

Since the matrix of the sample has changed after enzymatic hydrolysis, it is necessary to perform another methodological examination of the enzymatically hydrolyzed sample. The UHPLC-MS/MS method also validated linearity, specificity, LLOQ, precision, stability, matrix effect and recovery as the method validation of prior-enzymolysis sample except that all the samples were hydrolyzed by sulfatase. And the chromatograms and data are listed in Figure S4, S5 and Table S6-10.


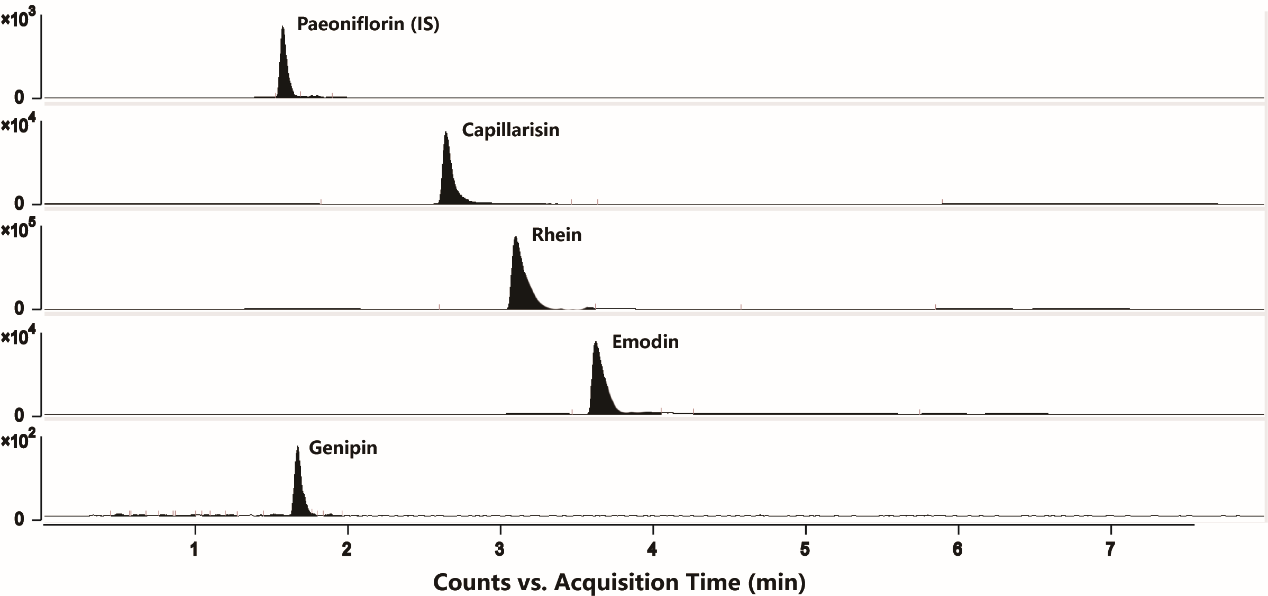


**Figure S4** Multiple chromatograms of four analytes and internal standard.


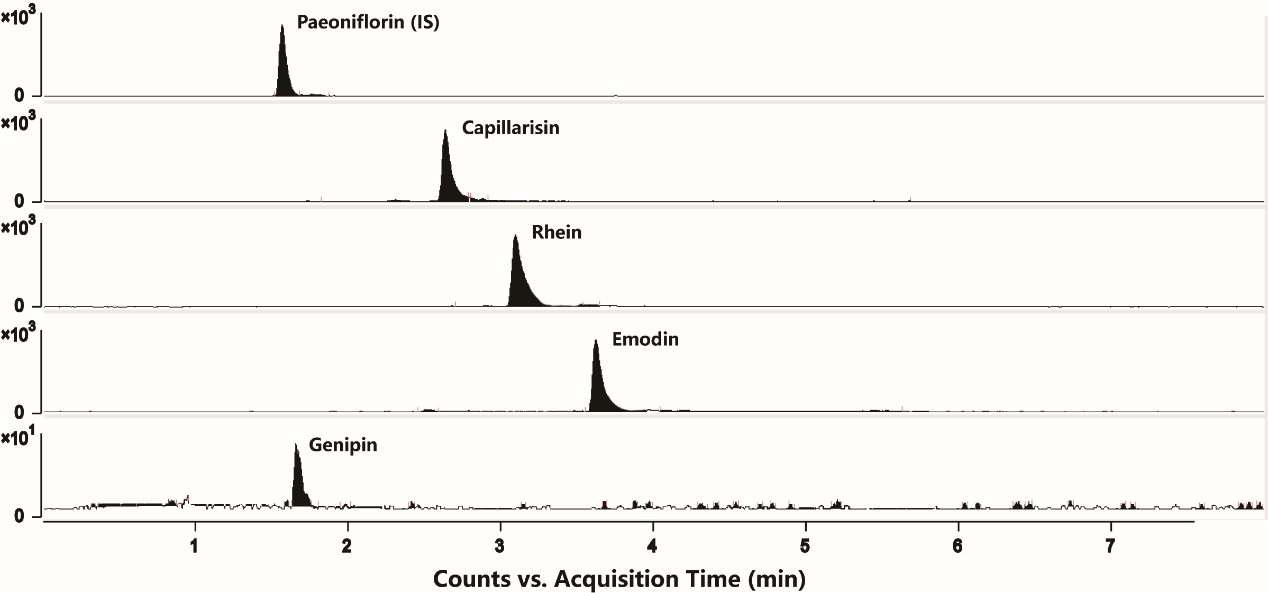


**Figure S5** Multiple chromatograms of four components and internal standard in plasma.

**TABLE S6** The Linearity Data, Calibration Range and LLOQ of Four Compounds.

| Component | Calibration Range  (ng/mL) | R^2^ | Calibration Range (ng/mL) | LLOQ  (ng/mL) |
| --- | --- | --- | --- | --- |
| Capillarisin | Y=0.030727*X+1.001656 | 0.9926 | 2.07-4240 | 2.07 |
| Rhein | Y=0.051133*X-0.148113 | 0.9912 | 2.07-4880 | 2.07 |
| Emodin | Y=0.062991*X-0.009269 | 0.9913 | 2.38-4240 | 2.38 |
| Genipin | Y=0.000643*X+0.003861 | 0.9916 | 2.15-4400 | 2.15 |

**TABLE S7** Intra- and Inter-day Precision and Accuracy of the Assay

| Intra-Day (n=5) | | | | | | | | | | |
| --- | --- | --- | --- | --- | --- | --- | --- | --- | --- | --- |
|  | Added | Founded | RSD | Accuracy |  | Added | Founded | RSD | Accuracy |  |
|  | (ng/mL) | (ng/mL) | (%) | (RE %) |  | (ng/mL) | (ng/mL) | (%) | (RE %) |  |
| Em | 9.53 | 9.84±0.50 | 5.1 | 3.21 | Gep | 8.59 | 8.19±0.58 | 7.1 | -4.72 |  |
|  | 76.25 | 76.63±5.51 | 7.2 | 0.50 |  | 68.75 | 68.09±7.54 | 11.1 | -0.95 |  |
|  | 2440 | 2297±127.3 | 5.5 | -5.87 |  | 2200 | 2470±56.14 | 2.3 | 12.25 |  |
| Rh | 8.28 | 7.51±0.41 | 5.4 | -9.37 | Cap | 8.28 | 7.98±0.64 | 8.0 | -3.61 |  |
|  | 66.25 | 60.55±0.95 | 1.6 | -8.61 |  | 66.25 | 64.29±2.11 | 3.3 | -2.96 |  |
|  | 2120 | 2251±92.58 | 4.1 | 6.20 |  | 2120 | 2352±67.36 | 2.9 | 10.95 |  |

| Inter-Day (n=5 series per day, for 3 days) | | | | | | | | | |
| --- | --- | --- | --- | --- | --- | --- | --- | --- | --- |
|  | Added | Founded | RSD | Accuracy |  | Added | Founded | RSD | Accuracy |
|  | (ng/mL) | (ng/mL) | (%) | (RE %) |  | (ng/mL) | (ng/mL) | (%) | (RE %) |
| Em | 9.53 | 10.11±0.27 | 2.7 | 6.10 | Gep | 8.59 | 8.62±0.56 | 6.5 | 0.27 |
|  | 76.25 | 76.88±4.85 | 6.3 | 0.83 |  | 68.75 | 66.89±5.07 | 7.6 | -2.71 |
|  | 2440 | 2276±40.62 | 1.8 | -6.71 |  | 2200 | 2409±106.7 | 4.4 | 9.52 |
| Rh | 8.28 | 8.45±0.82 | 9.8 | 2.05 | Cap | 8.28 | 8.19±0.29 | 3.6 | -1.07 |
|  | 66.25 | 60.54±1.26 | 2.1 | -8.62 |  | 66.25 | 63.14±1.00 | 1.6 | -4.69 |
|  | 2120 | 2161±243.7 | 11.3 | 1.92 |  | 2120 | 2323±70.79 | 3.0 | 9.58 |

Cap, Capillarisin; Gep, Genipin; Rh, Rhein; Em, Emodin;

**TABLE S8** Matrix Effects for the Detection of Four Compounds (n = 5).

|  | Concentration | ME | RSD |  | Concentration | ME | RSD |
| --- | --- | --- | --- | --- | --- | --- | --- |
|  | (ng/mL) | (%) | (%) |  | (ng/mL) | (%) | (%) |
| Em | 9.53 | 66.4 | 10.8 | Gep | 8.59 | 76.9 | 6.7 |
|  | 76.25 | 65.8 | 9.8 |  | 68.75 | 78.9 | 7.0 |
|  | 2440 | 77.8 | 2.3 |  | 2200 | 76.5 | 2.5 |
| Rh | 8.28 | 83.8 | 9.5 | Cap | 8.28 | 84.7 | 12.8 |
|  | 66.25 | 78.0 | 2.8 |  | 66.25 | 106.5 | 10.3 |
|  | 2120 | 79.8 | 1.9 |  | 2120 | 85.9 | 3.4 |

**TABLE S9** Recoveries for the Detection of Four Compounds (n = 5).

|  | Concentration | RE | RSD |  | Concentration | RE | RSD |
| --- | --- | --- | --- | --- | --- | --- | --- |
|  | (ng/mL) | (%) | (%) |  | (ng/mL) | (%) | (%) |
| Em | 9.53 | 58.0 | 13.4 | Gep | 8.59 | 60.8 | 12.3 |
|  | 76.25 | 53.4 | 2.3 |  | 68.75 | 64.1 | 13.6 |
|  | 2440 | 51.9 | 5.5 |  | 2200 | 60.3 | 4.0 |
| Rh | 8.28 | 60.6 | 6.0 | Cap | 8.28 | 64.7 | 3.6 |
|  | 66.25 | 45.4 | 4.2 |  | 66.25 | 54.6 | 4.5 |
|  | 2120 | 46.5 | 3.2 |  | 2120 | 66.4 | 3.9 |

**TABLE S10** Stability of the Four Compounds.

|  |  | Accuracy (RE %) | | | |
| --- | --- | --- | --- | --- | --- |
| Compound | Concentration (ng/mL) | Post-Preparative  4℃,for 24 h | Short-Term  25℃for 24 h | Freezing-Thawing  -70℃, 3 cycles | Long-Term  -20℃, 2 months |
| Em | 9.53 | 100.6 | 99.99 | 100.6 | 107.2 |
|  | 76.25 | 92.18 | 88.37 | 94.92 | 111.4 |
|  | 2440 | 105.1 | 91.10 | 99.34 | 107.4 |
| Rh | 8.28 | 111.4 | 92.42 | 98.58 | 110.2 |
|  | 66.25 | 98.90 | 91.29 | 90.11 | 101.7 |
|  | 2120 | 109.0 | 95.88 | 109.5 | 103.1 |
| Gep | 8.59 | 95.17 | 102.1 | 99.20 | 109.6 |
|  | 68.75 | 93.45 | 95.53 | 94.88 | 91.49 |
|  | 2200 | 111.4 | 108.2 | 106.6 | 98.69 |
| Cap | 8.28 | 94.65 | 105.2 | 104.0 | 99.69 |
|  | 66.25 | 93.08 | 89.26 | 95.81 | 105.2 |
|  | 2120 | 105.8 | 109.4 | 107.2 | 104.7 |

**2.2 Application of Pharmacokinetics Study of Active Components of Yinchenhao Decoction in Rats**

We utilized Das 2.1 to get pharmacokinetic parameters of active constituents and phase II metabolites of YCHD in rats, and all the parameters are listed in Table S11.

**TABLE S11** Pharmacokinetic parameters of active constituents and phase II metabolites of YCHD in rats.

|  | **Rhein** | | **4-hydroxyacetophenone** | |
| --- | --- | --- | --- | --- |
|  | Control group | Model group | Control group | Model group |
| Cmax (ug/L) | 3041.6±397.4 | 422.1±87.5 | 1579.6±133.2 | 676.2±85.0 |
| Tmax (min) | 15±0 | 15±0 | 15±0 | 15±0 |
| t1/2z (min) | 63.6±0.5 | 131.3±48.7 | 829.4±817.5 | 1556.1±1260.1 |
| Ka (1/min) | 4443.6±454.1 | 478.1±200.1 | 406.1±333.9 | 202.7±235.9 |
| Ke (1/min) | 3043.5±397.7 | 422.3±70.6 | 1582.0±133.8 | 1005.2±587.1 |
| Vz/F (L/kg) | 3.793±0.53 | 41.4±20.8 | 0.167±0.014 | 428.737±286.662 |
| CLz/F (L/min/kg) | 0.041±0.006 | 0.221±0.084 | 190.9±176.8 | 0.215±0.037 |
| MRT (0-∞)(min) | 120.578±6.554 | 169.7±94.2 | 289.1±286.5 | 1113.7±1092.7 |
| AUC(0-∞) (ug/L*min) | 874792.3±503744.0 | 172453.1±108249.8 | 192358.2±102858.3 | 113028.5±56412.0 |

|  | **Geniposidic acid** | | **genipin-1-β-D-gentiobiodide** | |
| --- | --- | --- | --- | --- |
|  | Control group | Model group | Control group | Model group |
| Cmax (ug/L) | 449.0±34.2 | 1694.6±251.3 | 105.7±3.9 | 63.9±10.1 |
| Tmax (min) | 264±100.4 | 240±0.0 | 21±8.2 | 15±0.0 |
| t1/2z (min) | 193.6±45.3 | 313.8±86.2 | 115.5±32.3 | 168.4±180.8 |
| Ka (1/min) | 11255.3±11024.4 | 41280.6±37036.2 | 95.5±16.2 | 106.8±25.5 |
| Ke (1/min) | 449.3±34.2 | 665.8±85.0 | 105.8±3.9 | 64.0±10.1 |
| Vz/F (L/kg) | 8.6±3.2 | 4986.4±1184.2 | 170.3±52.3 | 279.9±254.4 |
| CLz/F (L/min/kg) | 0.03±0.004 | 11.3±2.3 | 1.044±0.056 | 1.26±0.165 |
| MRT(0-∞) (min) | 356.0±15.4 | 570.2±125.0 | 127.9±49.1 | 188.2±128.2 |
| AUC(0-∞) (ug/L*min) | 1374849.8±734718.1 | 3007701.6±1408367.9 | 38557.0±20175.1 | 31749.4±15408.0 |

|  | **Scandoside methyl ester** | | **Caffeic acid** | |
| --- | --- | --- | --- | --- |
|  | Control group | Model group | Control group | Model group |
| Cmax (ug/L) | 127.3±8.9 | 93.8±13.7 | 285.9±14.5 | 78.9±4.9 |
| Tmax (min) | 21±8.2 | 264±100.4 | 15±0.0 | 24±20.1 |
| t1/2z(min) | 229.3±105.6 | 688.1±196.6 | 56.3±43.0 | 80.1±27.5 |
| Ka (1/min) | 477.5±133.9 | 22.6±38.8 | 119.2±82.6 | 75.2±24.5 |
| Ke (1/min) | 147.8±52.0 | 121.1±38.6 | 286.3±14.7 | 79.0±4.9 |
| Vz/F (L/kg) | 62.0±30.8 | 95.6±26.6 | 35.7±29.1 | 132.0±39.7 |
| CLz/F (L/min/kg) | 0.184±0.033 | 0.098±0.016 | 0.445±0.038 | 1.189±0.095 |
| MRT(0-∞) (min) | 364.9±31.3 | 1003.7±235.1 | 85.3±12.1 | 103.0±22.3 |
| AUC(0-∞) (ug/L*min) | 233687.2±127559.1 | 300942.4±177466.0 | 63847.8±53249.0 | 32651.4±19640.7 |

|  | **Chlorogenic acid** | | **Geniposide** | |
| --- | --- | --- | --- | --- |
|  | Control group | Model group | Control group | Model group |
| Cmax (ug/L) | 33.0±4.5 | 78.9±4.9 | 524.0±59.4 | 577.6±59.6 |
| Tmax (min) | 15±0.0 | 27±12.6 | 18±6.7 | 27±6.7 |
| t1/2z (min) | 61729.4±135135.6 | 1657.7±2115.2 | 114.1±45.9 | 338.6±28.0 |
| Ka (1/min) | 19.0±16.288 | 38.945±18.022 | 702.9±413.6 | 1543.0±592.8 |
| Ke (1/min) | 129.634±220.0 | 23.6±3.0 | 535.1±47.2 | 577.8±59.6 |
| Vz/F (L/kg) | 3801.5±2062.6 | 2107.4±1661.3 | 21.1±12.0 | 22.4±7.5 |
| CLz/F (L/min/kg) | 1.484±0.921 | 1.361±0.523 | 0.122±0.022 | 0.045±0.012 |
| MRT (0-∞)(min) | 88391.9±194649.9 | 1988.8±2573.6 | 201.5±13.9 | 450.0±56.7 |
| AUC(0-∞)(ug/L*min) | 11559.2±5248.0 | 21437.2±12572.8 | 384279.8±184237.0 | 956445.5±420665.9 |

|  | **Phase II metabolites of rhein** | | **Phase II metabolites of genipin** | |
| --- | --- | --- | --- | --- |
|  | Control group | Model group | Control group | Model group |
| Cmax (ug/L) | 3111.4±1548.1 | 565.2±224.2 | 397.5±88.0 | 1139.2±130.6 |
| Tmax (min) | 40±23.2 | 15±0.0 | 15±0.0 | 54±8.2 |
| t1/2z (min) | 193.947±20.6 | 211.3±126.7 | 57.9±3.1 | 64.7±29.7 |
| Ka (1/min) | 24136.3±52459.0 | 2415.7±4197.7 | 874.8±1572.8 | 1543.0±592.8 |
| Ke (1/min) | 3778.6±2198.8 | 565.7±224.5 | 104.9±565.8 | 0.255±0.091 |
| Vz/F (L/kg) | 6.0±0.7 | 23.7±13.4 | 5.4±1.7 | 1.5±0.7 |
| CLz/F (L/min/kg) | 0.021±0.003 | 0.082±0.014 | 0.064±0.017 | 0.017±0.004 |
| MRT(0-∞) (min) | 194.7±22.1 | 382.8±122.8 | 271.1±21.6 | 341.8±60.6 |
| AUC(0-∞)(ug/L*min) | 1652391.1±995772.5 | 529535.4±245034.0 | 692818.0±439496.1 | 2582594.1±1308580.4 |

|  | **Phase II metabolites of emodin** | | **Phase II metabolites of capillarisin** | |
| --- | --- | --- | --- | --- |
|  | Control group | Model group | Control group | Model group |
| Cmax (ug/L) | 509.6±57.0 | 75.982±32.491 | 195.7±98.5 | ND |
| Tmax (min) | 27.0±16.4 | 24.0±13.4 | 5.0±0.0 | ND |
| t1/2z (min) | 176.3±21.4 | 691.2±199.8 | 159.0±159.7 | ND |
| Ka (1/min) | 5279.2±10436.9 | 1835.8±1361.1 | 2558.3±2351.0 | ND |
| Ke (1/min) | 533.5±96.6 | 76.0±32.6 | 196.3±99.0 | ND |
| Vz/F (L/kg) | 23.0±5.0 | 211.0±68.8 | 401.2±182.0 | ND |
| CLz/F (L/min/kg) | 0.09±0.016 | 0.21±0.022 | 2.3±1.1 | ND |
| MRT(0-∞) (min) | 308.0±29.4 | 951.7±292.4 | 302.7±92.2 | ND |
| AUC(0-∞)(ug/L*min) | 381836.2±203100.9 | 135757.8±68473.4 | 19339.4±12413.9 | ND |

**3. The detailed biochemical indicators of all the groups**

| **TABLE S12** Detail values of biochemical indices reflecting protective effect of YCHD against ANIT-induced cholestasis | | | | | |
| --- | --- | --- | --- | --- | --- |
| Group | ALT (IU/L) | AST (IU/L) | TBIL (μmol/L) | TBA (μmol/L) | DBIL (μmol/L) |
| Control | 57.8±7.7 | 142.0±20.3 | 0.9±0.3 | 36.2±23.7 | 0.2±0.1 |
| Model | 495.6±201.6^***^ | 670.2±212.1^***^ | 50.3±11.5^***^ | 619.2±68.2^***^ | 35.9±6.5^***^ |
| UDCA | 400.6±200.0 | 611.8±257.2 | 28.2±19.0^##^ | 428.1±55.9^###^ | 21.2±9.0^##^ |
| Low-dose | 356.0±100.3 | 520.0±115.8 | 53.4±10.9 | 510.9±84.5^#^ | 35.0±7.2 |
| Medium-dose | 293.8±104.4^#^ | 533.2±165.1 | 45.4±12.6 | 547.4±103.5 | 26.6±6.8^#^ |
| High-dose | 259.8±95.5^##^ | 436.6±80.0^#^ | 37.1±2.7^#^ | 516.7±58.5^#^ | 25.4±3.2^#^ |
| All data were represent as means ± S.D. ALT, alanine aminotransferase; AST, aspartate aminotransferase; TBIL, serum total bilirubin; TBA, total bile acid; DBIL, serum direct bilirubin. *** represents *p* < 0.001 compared with the control group, #, ## and ## represents *p* < 0.05, *p* < 0.01 and *p* < 0.001 compared with the RP-treated group, respectively. | | | | | |

**4. Determination of Metabolic Enzyme and Transporters Expression in Rat Liver by LC-MS/MS**

**4.1 Method Validation**


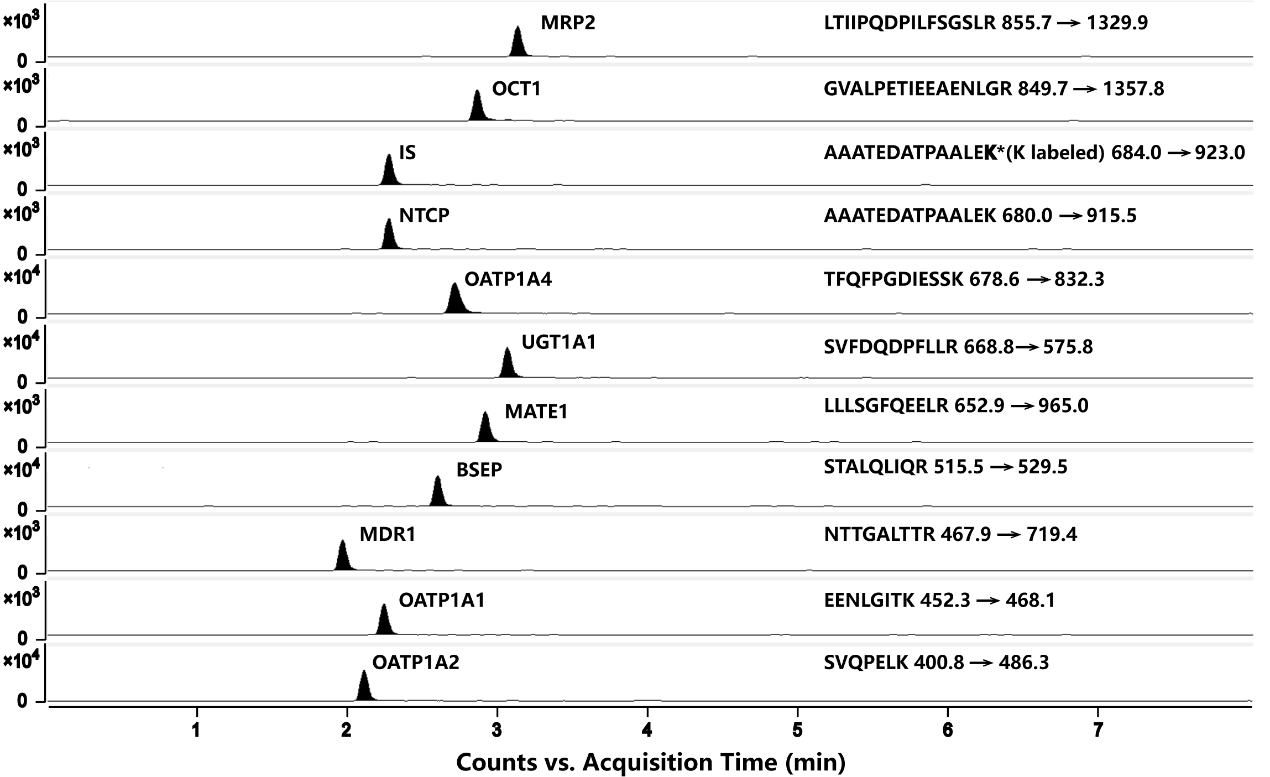
The UHPLC-MS/MS determination method of ten peptides in rat liver was validated followed instructions described in Section 2.5.3.4. Figure S6 presents the chromatograms of ten peptides. Table S13-S16 shows methodological study data of this quantitative method.

**Figure S6** Multiple chromatograms of ten peptides and internal standard (labelled peptide).

**TABLE S13** The Linearity Data, Calibration Range and LLOQ of Ten Peptides Analytes.

| Transporter | Signature Peptides | Calibration Range (nM) | R^2^ | Weighting Factor | LLOQ (nM) |
| --- | --- | --- | --- | --- | --- |
| Mrp2 | LTIIPQDPILFSGSLR | 0.254-32.53 | 0.9909 | 1/x^2^ | 0.023 |
| Oct1 | GVALPETIEEAENLGR | 0.221-28.26 | 0.9901 | 1/x^2^ | 0.155 |
| Ntcp | AAATEDATPAALEK | 0.378-48.33 | 0.9962 | 1/x^2^ | 0.141 |
| Oatp1a4 | TFQFPGDIESSK | 0.311-39.87 | 0.9939 | 1/x^2^ | 0.098 |
| Mate1 | LLLSGFQEELR | 0.363-46.46 | 0.9934 | 1/x^2^ | 0.326 |
| Bsep | STALQLIQR | 0.433-55.45 | 0.9917 | 1/x^2^ | 0.245 |
| Mdr1 | NTTGALTTR | 0.452-57.86 | 0.9907 | 1/x^2^ | 0.010 |
| Oatp1a1 | EENLGITK | 0.459-58.73 | 0.9906 | 1/x^2^ | 0.398 |
| Oatp1a2 | SVQPELK | 0.552-70.72 | 0.9902 | 1/x^2^ | 0.182 |
| Ugt1a1 | SVFDQDPFLLR | 0.342-43.84 | 0.9932 | 1/x^2^ | 0.100 |

**TABLE S14** Intra- and Inter-day Precision and Accuracy of the Peptide Analytes.

| Intra-Day (n=5) | | | | | | | | | |
| --- | --- | --- | --- | --- | --- | --- | --- | --- | --- |
|  | Added | Founded | RSD | Accuracy |  | Added | Founded | RSD | Accuracy |
|  | (nM) | (nM) | (%) | (RE %) |  | (nM) | (nM) | (%) | (RE %) |
| LTII | 0.508 | 0.464±0.061 | 13.2 | -8.8 | STAL | 0.857 | 0.857±0.129 | 15.0 | -1.0 |
|  | 4.066 | 3.987±0.524 | 13.2 | -1.9 |  | 7.644 | 7.644±0.707 | 9.3 | 10.3 |
|  | 16.26 | 14.70±0.945 | 6.4 | -9.6 |  | 26.69 | 26.69±1.975 | 7.4 | -3.7 |
| GVAL | 0.442 | 0.498±0.041 | 8.2 | 12.9 | NTTG | 0.853 | 0.853±0.051 | 6.0 | -5.6 |
|  | 3.532 | 3.703±0.461 | 12.4 | 4.8 |  | 7.660 | 7.660±0.943 | 12.3 | 5.9 |
|  | 14.13 | 13.39±1.124 | 8.4 | -5.3 |  | 28.26 | 28.26±1.188 | 4.2 | -2.3 |
| AAAT | 0.755 | 0.695±0.096 | 13.8 | -8.0 | EENL | 0.995 | 0.995±0.155 | 15.6 | 8.5 |
|  | 6.042 | 6.220±0.709 | 11.4 | 2.9 |  | 8.281 | 8.281±0.682 | 8.2 | 12.8 |
|  | 24.17 | 23.68±0.694 | 2.9 | -2.0 |  | 28.76 | 28.76±2.443 | 8.5 | -2.1 |
| TFQF | 0.623 | 0.699±0.080 | 11.5 | 12.2 | SVQP | 1.153 | 1.153±0.140 | 12.2 | 4.4 |
|  | 4.984 | 5.117±0.136 | 2.7 | 2.7 |  | 9.840 | 9.840±0.522 | 5.3 | 11.3 |
|  | 19.93 | 18.68±0.811 | 4.3 | -6.3 |  | 34.77 | 34.77±2.420 | 7.0 | -1.7 |
| LLLS | 0.726 | 0.613±0.021 | 3.5 | -15.6 | SVFD | 0.651 | 0.651±0.076 | 11.7 | -5.0 |
|  | 5.808 | 5.324±0.759 | 14.3 | -8.3 |  | 5.532 | 5.532±0.334 | 6.0 | 1.0 |
|  | 23.23 | 21.20±1.171 | 5.5 | -8.7 |  | 20.91 | 20.91±0.639 | 3.1 | -4.6 |

| Inter-Day (n=5) | | | | | | | | | |
| --- | --- | --- | --- | --- | --- | --- | --- | --- | --- |
|  | Added | Founded | RSD | Accuracy |  | Added | Founded | RSD | Accuracy |
|  | (nM) | (nM) | (%) | (RE %) |  | (nM) | (nM) | (%) | (RE %) |
| LTII | 0.508 | 0.526±0.055 | 10.4 | 3.5 | STAL | 0.857 | 0.825±0.047 | 5.7 | -4.8 |
|  | 4.066 | 4.040±0.068 | 1.7 | -0.6 |  | 7.644 | 6.763±0.794 | 11.7 | -2.4 |
|  | 16.26 | 15.03±0.790 | 5.3 | -7.6 |  | 26.69 | 26.57±0.868 | 3.3 | -4.2 |
| GVAL | 0.442 | 0.479±0.025 | 5.2 | 8.5 | NTTG | 0.853 | 0.855±0.018 | 2.0 | -5.5 |
|  | 3.532 | 3.780±0.280 | 7.4 | 7.0 |  | 7.660 | 7.319±0.351 | 4.8 | 1.2 |
|  | 14.13 | 15.96±2.667 | 16.7 | 13.0 |  | 28.26 | 27.95±1.918 | 6.9 | -3.4 |
| AAAT | 0.755 | 0.755±0.055 | 7.2 | -0.1 | EENL | 0.995 | 0.956±0.045 | 4.7 | 4.2 |
|  | 6.042 | 5.778±0.507 | 8.8 | -4.4 |  | 8.281 | 7.767±0.446 | 5.7 | 5.8 |
|  | 24.17 | 23.45±1.160 | 4.9 | -3.0 |  | 28.76 | 27.67±2.442 | 8.8 | -5.8 |
| TFQF | 0.623 | 0.644±0.076 | 11.7 | 3.4 | SVQP | 1.153 | 1.131±0.030 | 2.7 | 2.4 |
|  | 4.984 | 5.098±0.092 | 1.8 | 2.3 |  | 9.840 | 9.384±0.453 | 4.8 | 6.2 |
|  | 19.93 | 19.12±0.719 | 3.8 | -4.1 |  | 34.77 | 34.33±3.210 | 9.4 | -2.9 |
| LLLS | 0.726 | 0.683±0.063 | 9.3 | -5.9 | SVFD | 0.651 | 0.651±0.027 | 4.1 | -4.9 |
|  | 5.808 | 5.759±0.461 | 8.0 | -0.8 |  | 5.532 | 5.567±0.174 | 3.1 | 1.6 |
|  | 23.23 | 23.13±1.707 | 7.4 | -0.4 |  | 20.91 | 20.88±1.224 | 5.9 | -4.7 |

**TABLE S15** Recoveries of the Ten Peptides Analytes.

|  | Initial  (nM) | Added  (nM) | Detected  (nM) | Recovery  (%) | SD  (nM) | RSD  (%) |
| --- | --- | --- | --- | --- | --- | --- |
| AAAT | 7.160 | 5.771 | 13.91 | 116.9 | 0.469 | 3.4 |
|  |  | 7.214 | 14.38 | 100.1 | 0.543 | 3.8 |
|  |  | 8.657 | 16.04 | 102.6 | 2.445 | 15.2 |
| LTII | 3.102 | 2.606 | 6.146 | 116.8 | 0.683 | 11.1 |
|  |  | 3.258 | 7.235 | 126.9 | 0.092 | 1.3 |
|  |  | 3.909 | 6.495 | 86.8 | 0.362 | 5.6 |
| SVFD | 8.805 | 7.163 | 16.29 | 104.5 | 2.142 | 13.1 |
|  |  | 8.954 | 17.99 | 102.6 | 2.477 | 13.8 |
|  |  | 10.74 | 16.00 | 67.0 | 2.793 | 17.5 |
| EENL | 3.543 | 2.916 | 6.115 | 88.2 | 0.905 | 14.8 |
|  |  | 3.645 | 8.860 | 145.9 | 0.636 | 7.2 |
|  |  | 4.374 | 7.384 | 87.8 | 0.610 | 8.3 |
| GVAL | 1.857 | 1.484 | 3.868 | 135.5 | 0.264 | 6.8 |
|  |  | 1.855 | 4.124 | 122.2 | 0.489 | 11.9 |
|  |  | 2.226 | 4.495 | 118.5 | 0.737 | 16.4 |
| LLLS | 1.045 | 0.889 | 2.106 | 119.4 | 0.149 | 7.1 |
|  |  | 1.112 | 2.570 | 137.2 | 0.050 | 1.9 |
|  |  | 1.334 | 2.782 | 130.3 | 0.361 | 13.0 |
| NTTG | 1.056 | 0.899 | 1.835 | 79.0 | 0.296 | 16.1 |
|  |  | 1.124 | 2.079 | 84.9 | 0.496 | 10.3 |
|  |  | 1.349 | 2.968 | 136.7 | 0.296 | 10.0 |
| STAL | 2.645 | 2.189 | 5.199 | 116.7 | 0.129 | 2.5 |
|  |  | 2.737 | 6.208 | 130.2 | 0.155 | 2.5 |
|  |  | 3.284 | 5.594 | 89.8 | 0.552 | 9.9 |
| SVQP | 2.307 | 2.125 | 5.722 | 160.6 | 0.203 | 3.6 |
|  |  | 2.657 | 6.270 | 149.1 | 0.595 | 9.5 |
|  |  | 3.188 | 5.790 | 109.2 | 0.096 | 1.7 |
| TFQF | 1.812 | 1.530 | 3.741 | 126.1 | 0.134 | 3.6 |
|  |  | 1.912 | 3.970 | 112.8 | 0.202 | 5.1 |
|  |  | 2.294 | 3.698 | 82.2 | 0.082 | 2.2 |

**TABLE S16** Stability of the Ten Peptides Analytes.

|  |  | Accuracy (RE %) | | | |
| --- | --- | --- | --- | --- | --- |
| Compound | Concentration (nM) | Post-Preparative  4℃,for 24 h | Short-Term  25℃for 24 h | Freezing-Thawing  -20℃, 3 cycles | Long-Term  -20℃, 2 months |
| LTII | 0.508 | 102.2 | 90.2 | 96.4 | 90.0 |
|  | 4.066 | 82.9 | 112.1 | 98.4 | 100.1 |
|  | 16.26 | 115.0 | 102.0 | 96.5 | 91.0 |
| GVAL | 0.442 | 98.4 | 93.0 | 90.6 | 106.8 |
|  | 3.532 | 90.2 | 100.7 | 101.0 | 97.2 |
|  | 14.13 | 103.1 | 99.1 | 100.0 | 104.7 |
| AAAT | 0.755 | 104.8 | 109.3 | 88.2 | 90.5 |
|  | 6.042 | 102.4 | 99.0 | 96.0 | 99.4 |
|  | 24.17 | 87.9 | 98.3 | 97.3 | 106.7 |
| TFQF | 0.623 | 100.1 | 100.9 | 98.3 | 84.6 |
|  | 4.984 | 92.3 | 98.8 | 95.4 | 97.9 |
|  | 19.93 | 101.2 | 96.7 | 97.1 | 109.4 |
| LLLS | 0.726 | 92.7 | 115.8 | 90.4 | 106.7 |
|  | 5.808 | 98.3 | 111.1 | 100.1 | 98.5 |
|  | 23.23 | 110.6 | 99.5 | 102.6 | 104.9 |
| STAL | 0.857 | 94.4 | 88.6 | 108.2 | 102.6 |
|  | 7.644 | 101.9 | 93.1 | 91.1 | 103.7 |
|  | 26.69 | 102.8 | 102.3 | 88.1 | 104.3 |
| NTTG | 0.853 | 91.4 | 100.3 | 104.2 | 90.0 |
|  | 7.660 | 97.4 | 107.9 | 90.8 | 106.3 |
|  | 28.26 | 98.0 | 99.3 | 94.2 | 116.8 |
| EENL | 0.995 | 108.8 | 100.6 | 112.0 | 99.3 |
|  | 8.281 | 98.6 | 104.4 | 100.2 | 98.6 |
|  | 28.76 | 101.9 | 99.8 | 101.0 | 96.5 |
| SVQP | 1.153 | 111.4 | 113.5 | 104.5 | 90.4 |
|  | 9.840 | 97.0 | 102.5 | 97.2 | 99.3 |
|  | 34.77 | 96.2 | 99.1 | 99.7 | 106.1 |
| SVFD | 0.651 | 110.9 | 99.4 | 102.5 | 103.4 |
|  | 5.532 | 94.5 | 107.2 | 98.2 | 99.0 |
|  | 20.91 | 97.1 | 100.5 | 104.7 | 107.0 |

**4.2 The values of expression of drug metabolic enzyme and protein transporters in all the group**

| **TABLE S17** Specific values of expression levels of drug metabolic enzyme and other nine protein transporters in rats liver | | | | | | | | | | |
| --- | --- | --- | --- | --- | --- | --- | --- | --- | --- | --- |
| Group | Ntcp  (fmol/μg protein) | Oatp1a1  (fmol/μg protein) | Oct1  (fmol/μg protein) | Mate1  (fmol/μg protein) | Mrp2  (fmol/μg protein) | Mdr1  (fmol/μg protein) | Bsep  (fmol/μg protein) | Ugt1a1  (fmol/μg protein) | Oatp1a2  (fmol/μg protein) | Oatp1a4  (fmol/μg protein) |
| Control | 8.27±2.40 | 4.76±0.86 | 2.26±0.77 | 2.56±1.05 | 3.32±0.52 | 1.27±0.53 | 21.2±3.66 | 8.35±1.00 | 1.52±0.19 | 1.58±0.27 |
| Model | 1.78±0.86^***^ | 2.68±1.11^**^ | 1.00±0.49^**^ | 1.81±1.75 | 1.38±0.59^***^ | 0.25±0.07^***^ | 14.0±2.85^**^ | 2.67±0.79^***^ | 0.78±0.32^**^ | 0.35±0.12^***^ |
| UDCA | 4.22±1.51^#^ | 3.42±1.23 | 2.22±0.59^##^ | 3.43±1.38 | 2.21±0.87 | 0.67±0.32^#^ | 18.1±2.56^#^ | 6.06±1.34^##^ | 1.27±0.18^#^ | 0.49±0.11 |
| Low | 3.99±1.38^#^ | 2.51±0.75 | 1.99±0.67^#^ | 2.83±1.04 | 3.28±0.43^###^ | 0.42±0.21 | 17.4±1.53 | 6.19±0.85^###^ | 0.91±0.14 | 0.74±0.29^#^ |
| Medium | 4.31±1.13^#^ | 3.21±0.83 | 2.36±0.66^##^ | 4.61±2.98 | 2.82±0.80^##^ | 0.57±0.19 | 18.8±2.28^#^ | 6.76±1.70^###^ | 1.25±0.50^#^ | 0.90±0.38^##^ |
| High | 3.84±1.40^#^ | 2.58±0.99 | 2.01±0.60^#^ | 6.76±2.84^##^ | 3.73±0.64^###^ | 0.52±0.15 | 23.7±3.83^###^ | 6.96±1.79^###^ | 1.41±0.35^##^ | 0.84±0.27^##^ |
| Expression levels were expressed as means ± S.D. Low, Medium and High represent rats treated with YCHD at dosages of 6.0, 9.0 and 12 g/kg, respectively. **, *** represent *p* < 0.01 and *p* < 0.001 compared with the control group, #, ## and ## represents *p* < 0.05, *p* < 0.01 and *p* < 0.001 compared with the ANIT-treated cholestasis group, respectively. | | | | | | | | | | |
